# Integrative prediction of gene expression with chromatin accessibility and conformation data

**DOI:** 10.1186/s13072-020-0327-0

**Published:** 2020-02-06

**Authors:** Florian Schmidt, Fabian Kern, Marcel H. Schulz

**Affiliations:** 1 High-throughput Genomics & Systems Biology, Cluster of Excellence on Multimodal Computing and Interaction, Saarland Informatics Campus, 66123 Saarbrücken, Germany; 2 Computational Biology & Applied Algorithmics, Max-Planck Institute for Informatics, Saarland Informatics Campus, 66123 Saarbrücken, Germany; 3 Center for Bioinformatics, Saarland Informatics Campus, 66123 Saarbrücken, Germany; 4 Genome Institute of Singapore, A*STAR, 60 Biopolis Street, Singapore, 138672 Singapore; 5 Chair for Clinical Bioinformatics, Saarland Informatics Campus, 66123 Saarbrücken, Germany; 6 Institute of Cardiovascular Regeneration, Goethe-University, Theodor-Stern-Kai 7, 60590 Frankfurt am Main, Germany; 7 German Center for Cardiovascular Research, Partner Site Rhein-Main, Theodor-Stern-Kai 7, 60590 Frankfurt am Main, Germany

**Keywords:** Machine learning, Chromatin accessibility, DNase1-seq, Chromatin conformation, Gene regulation, HiC, HiChIP, Gene expression prediction

## Abstract

**Background:**

Enhancers play a fundamental role in orchestrating cell state and development. Although several methods have been developed to identify enhancers, linking them to their target genes is still an open problem. Several theories have been proposed on the functional mechanisms of enhancers, which triggered the development of various methods to infer promoter–enhancer interactions (PEIs). The advancement of high-throughput techniques describing the three-dimensional organization of the chromatin, paved the way to pinpoint long-range PEIs. Here we investigated whether including PEIs in computational models for the prediction of gene expression improves performance and interpretability.

**Results:**

We have extended our $\textsc{TEPIC}$ framework to include DNA contacts deduced from chromatin conformation capture experiments and compared various methods to determine PEIs using predictive modelling of gene expression from chromatin accessibility data and predicted transcription factor (TF) motif data. We designed a novel machine learning approach that allows the prioritization of TFs binding to distal loop and promoter regions with respect to their importance for gene expression regulation. Our analysis revealed a set of core TFs that are part of enhancer–promoter loops involving YY1 in different cell lines.

**Conclusion:**

We present a novel approach that can be used to prioritize TFs involved in distal and promoter-proximal regulatory events by integrating chromatin accessibility, conformation, and gene expression data. We show that the integration of chromatin conformation data can improve gene expression prediction and aids model interpretability.

## Introduction

Understanding the processes involved in gene regulation is an important endeavour in computational biology. Key players in gene regulation are transcription factors (TFs), DNA binding proteins that are essential in regulating transcriptional processes. They are important in establishing and maintaining cellular identity and their dysfunction is related to several diseases [1].

TFs bind to promoters of genes, which are in close proximity to their transcription start site (TSS) and to enhancers, regulatory regions that can be several thousand base pairs away from the regulated gene [2]. Since enhancers have been described for the first time in 1981 by Banerji et al. [3], numerous studies shed light on their functional role.

For example, enhancers were shown to be essential in cell differentiation [4]. Also, it has been reported that mutations occurring in enhancer regions, can not only lead to changes in gene expression [5, 6], but can also increase the probability to contract certain diseases, for instance *Hirschsprung’s disease* [7]. These effects are likely to be caused by an altered binding of TFs due to SNPs occurring in enhancer sequences [2, 8, 9]. To understand the function of enhancers, a crucial step after identification of putative enhancer regions is to link them to their target genes.

Recently, considerable progress has been made in identifying putative enhancer regions: In the past decade, many epigenetic data sets have been generated in consortia like ENCODE [10], Blueprint [11] and Roadmap [12]. Histone Modifications, especially *H3K27ac* and *H3K4me1*, have been used in unsupervised computational approaches, such as $\textsc{CHROMHMM}$ [13], $\textsc{EPICSEG}$ [14], or $\textsc{REPTILE}$ [15] to highlight putative enhancer regions genome-wide.

Also (semi-)supervised methods, e.g. $\textsc{ENHANCER}$ [16], $\textsc{ENHANCERDBN}$ [17], or $\textsc{DECRES}$ [18], relying on experimentally validated enhancer regions used as training data have been proposed. Furthermore, it was shown that DNase-hypersensitive sites (DHSs) are good candidate sites for TF-binding [19, 20] and that DNase1-seq signal is also predictive for gene expression [20, 21]. Thus DHS sites, which are not located nearby promoters can be considered as candidate enhancer regions. However, it is still a fundamental biological question how enhancers interact with their potentially distantly located target genes. The most prevalent hypothesis is that enhancers are brought to close proximity to their target genes by chromosomal re-organization and DNA-looping. This hypothesis is known as the *looping* model. It is opposing the so-called *scanning* model, which states that an enhancer is usually regulating only its nearest active promoter [22]. Experimental evidence could be found for both models [2], hence it is likely that both mechanisms are occurring in nature.

Inspired by these models, several experimental and computational methods have been proposed to link enhancers to their target genes. Following the *scanning* model, two approaches are common in the field: (1) *window*-based linkage and (2) *nearest gene* linkage. In the window-based approach, a gene is associated with regulatory regions that are located within a defined genomic region around this gene [23, 24]. Alternatively, in the nearest gene approach, an enhancer is only associated with its nearest gene [25]. To reduce false-positive assignments, the *nearest gene* linkage is also often coupled to a correlation test between epigenetic signals in the enhancer and the expression of the candidate gene [26].

While approaches like $\textsc{JEME}$ [27], $\textsc{FOCS}$ [28] or $\textsc{STITCHIT}$ [29] offer the linkage of regulatory elements on a gene-specific level, these methods require the availability of large data sets for the considered species and tissues, which is generally not the case. In practice, the established *window* and *nearest gene*-based linkage paradigms are still being used [25]. However, the drawback of those approaches is that they do not include long-range enhancer–gene interactions, as proposed by the *looping* model. These have been experimentally determined using, for example fluorescence in situ hybridization (FISH), via the identification of enhancer RNAs (eRNAs) and their correlation to target genes, or via 3C-based high-throughput methods, for instance HiC, Capture-HiC, and HiChIP [30]. Especially the development of such high-throughput methods to analyse the 3D organization of the genome enables us to determine genome-wide DNA contacts [31]. Detailed analyses of individual genes, e.g. the $\beta$-*globin gene* showed that multiple contacts occur simultaneously at one genomic loci and also overlap with DHSs [32]. It was shown that loops are established by Cohesin, Mediator complexes and CTCF, which is known to act as an insulator protein. By performing genome-wide chromatin conformation capture experiments, it is possible to segment the genome in multiple topological associating domains (TADs). Also, it is known that there is more intra-TAD interaction among genes and enhancers than between TADs [33]. To mine the variety of data types that inform on chromatin structure and to establish promoter–enhancer interactions (PEIs) following the *looping* model, a wealth of tools have been published. The $\textsc{JEME}$ method by Cao et al. for instance is a two-level learning algorithm utilizing linear regression and random forest (RF) models that not only links candidate enhancers, derived from HMs, eRNAs and/or sites of ac $\textsc{JEME}$cessible chromatin, to their target genes, it also predicts sample-specific activity of the enhancers. In this process, is able to consider data on long-range interactions, e.g. ChIA-PET data [27]. A different approach has been taken by He et al. [34], in which a gold standard set of PEIs derived from ChIA-PET data are used to train classifiers to predict enhancer–promoter pairs. The classifiers consider various HMs, TF motif scores as well as sequence conservation. The approach taken by $\textsc{TARGETFINDER}$, suggested by Whalen et al., is yet another way to tackle the PEI inference problem [35]. They construct a gold standard set of active PEIs by combining chromatin state segmentations for ENCODE and Roadmap data sets with several HiC data sets. Using several genomic features such as DNA methylation, HMs, CAGE data, and binding information of proteins (e.g. Cohesin), they learn ensemble models that predict PEI interactions across cell-types. A general overview on available methods is provided in Yao et al. [2].

Despite the availability of PEI data derived from chromatin conformation data, it has not yet been integrated into computational methods inferring gene expression using experimentally or computationally determined TF binding events. Because of the tissue specificity of enhancers, including PEIs might augment the interpretability of such models and thereby lead to novel biological insights. Here, as a follow-up to our previous investigations [24, 36], we introduce an extension of the $\textsc{TEPIC}$ framework to account for PEIs inferred from chromatin conformation capture experiments or computational approaches. In other words, we do not propose a novel method to establish PEIs, but investigate whether PEIs inferred from chromatin conformation capture data can be meaningfully utilized in gene expression modelling. The usage of gene expression prediction models is established in literature, especially to highlight key cell-type-specific regulatory factors [24, 37, 38]. Generally, various sets of features have been proposed, e.g. TF ChIP-seq data [23], chromatin accessibility and predicted TF binding sites (TFBS) [25, 39–41], and the abundance of HMs [42]. Aside from traditional machine learning approaches such as linear regression or support vector regression, also deep learning models have been proposed in literature [43].

As a baseline, we illustrate using gene expression prediction that both *window* and *nearest gene* annotation approaches are not well suited to account for regulatory activity across the entire genome. Our new $\textsc{TEPIC}$ module extends a promoter-centric window by including far away genomic loci deduced from HiC and HiChIP data. While both HiC and HiChIP interactions improve gene expression prediction models, we observe a greater improvement with HiChIP data, an effect for which we outline several reasons. Furthermore, we illustrate that a distinct consideration of TF binding events in promoter and potentially far away enhancer regions allows for a fine-grained interpretation and analysis of transcriptional regulation through TFs.

## Materials and methods

### Data and preprocessing

In this study, we used gene expression quantified from RNA-seq data and DNase1-seq data for the cell lines K562, GM12878, IMR90, HUVEC, HCT116, Jurkat, and HeLa. All data are obtained from ENCODE, corresponding accession numbers are provided in Additional file 1: Table S1. Except for Jurkat, where gene expression estimates were quantified with $\textsc{SALMON}$ (version 0.8.2) using default parameters, gene expression estimates were directly downloaded from ENCODE. DHS sites have been identified using the peak caller $\textsc{JAMM}$ [44] (version 1.0.7.2), with default parameters configured. All peaks passing the automated filtering of $\textsc{JAMM}$ are considered. Additional file 1: Table S2 lists the number of identified peaks per cell line.

Furthermore, we obtained HiC data for K562, GM12878, IMR90, HUVEC, and HeLa from Rao et al. [31]. Specifically, we used the loop files as provided by the Lieberman-Aiden group, which were extracted from raw HiC contact matrices using the HiCCUPS algorithm [31]. In case of the HiC data sets used in this work the loops are of $5\;\text {kb}$, $10\;\text {kb}$, and $25\;\text {kb}$ resolution, respectively. A loop is defined as a pair of genomic loci that are in arbitrary genomic distance from each but, at the same time, are in close spatial proximity. In the following, the HiC resolution called *All* refers to loops of an arbitrary resolution, as this corresponds to a more conservative approach where we collect all available loops. For reasons of simplicity, inter-chromosomal loops, which resemble a less frequent type of contacts, are excluded. Additional file 1: Table S3 provides an overview on the HiC data considered in this work.

Additionally, we use processed HiChIP data (Additional file 1: Table S4) in which the TF YY1 was targeted in Jurkat, HCT116, and K562 cells generated by Weintraub et al. [45]. The data has a resolution of $5\,\text {kb}$. All data were obtained for the hg19 reference genome using gene annotation version 19 from GENCODE [46]. Each HiChIP interaction is assigned the number of paired-end tags (PET) that connect the two interacting loci (PET count). Furthermore, Weintraub et al. computed a significance value for each putative interaction (*q*-value) using a semi-Bayesian two-component mixture model [45].

We obtained chromatin state segmentations, containing 15 states generated with ChromHMM [13], for K562, GM12878, IMR90, HUVEC, and HeLa from ENCODE. As there was no ChromHMM annotation available for Jurkat, we approximate this using a Roadmap ChromHMM track for CD4+ CD25− Th Primary Cells (E043). We focus on the promoter states *TssA*(1) and *TssAFlnk*(2), as well as on the enhancer states *EnhG*(6), *Enh*(7), *BivFlnk*(11), and *EnhBiv*(12). ENCODE accession numbers are provided in Additional file 1: Table S1.

### Aggregation of promoter–enhancer interactions (PEIs) to the gene level

**Fig. 1:**
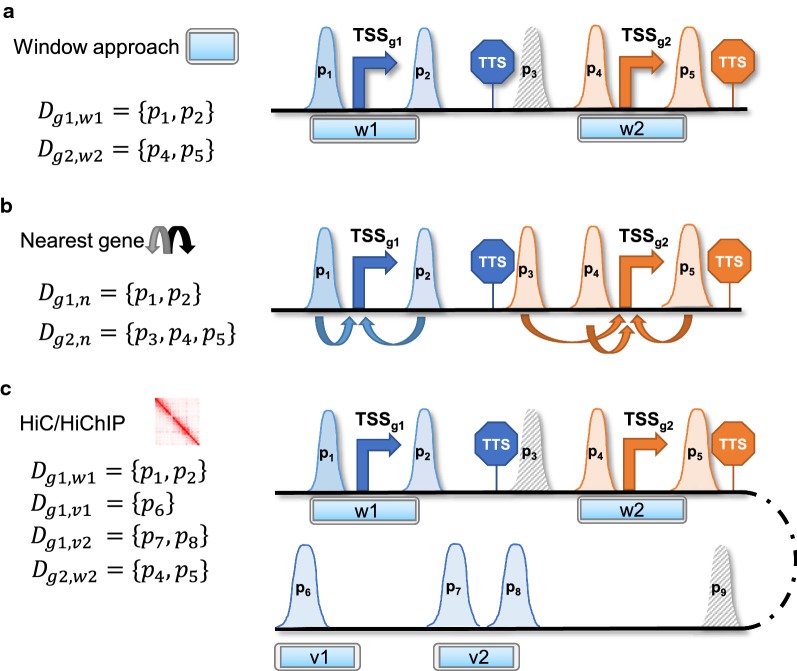
Assignment of DNase1-seq peaks to genes. The different setups are illustrated for two genes *g*1 and *g*2. The colour code of peaks and the border colour of segments indicate to which gene a peak is assigned. Peaks with a striped filling are not assigned to any gene. **a** In a window-based annotation, peaks are linked to a gene if they are located within a window *w* centred at the 5′ transcription start site (TSS) of a gene of interest. $D_{g1,w1}$ denotes the set of all DHSs overlapping window *w*1 centred around the promoter of gene *g*1. **b** Peaks are linked to the nearest gene, defining nearest as the gene with the closest TSS in linear genomic distance. Here, $D_{g1,n}$ refers to the set of all DHSs linked to gene *g*1 following the nearest gene approach. **c** Using HiC or HiChIP, secondary windows $v_i$ covering the distal regions linked to the TSS are considered in addition to the TSS window. For gene *g*1, two additional windows, *v*1 and *v*2, are considered, yielding the additional peak sets $D_{g1,v1}$ and $D_{g1,v2}.$

We apply three different strategies to aggregate PEIs from DNase1-seq data: (1) a window-based annotation, (2) a nearest gene-based linkage and (3) a window-based annotation that incorporates HiC or HiChIP data. An illustration of the PEI linkage methods is shown in Fig. 1.

We use the following notation throughout the article: considering a DHS site $d \in {\mathcal {D}}$, where ${\mathcal {D}}$ is the set of all DHS sites, we denote the length of *d* with *l*(*d*) and the DNase1-seq signal in *d* with *s*(*d*). We aggregate neighbouring genomic positions, which are assigned the same chromatin state from ChromHMM into one *segmentm*, representing a distinct ChromHMM state. The set of all considered segments is denoted with ${\mathcal {M}}$.

Here, we compute three different features for each gene *g*: (1) total peak length $pl_g$, (2) summarized peak count $pc_g$, and (3) aggregated peak signal $ps_g$ [36]:

1 $$\begin{aligned} pl_g&= \sum _{d \in {\mathcal {D}}_g} l(d)e^{-\frac{\mathrm{{dist}}(d,g)}{d_0}}, \end{aligned}$$

2 $$\begin{aligned} pc_g&= \sum _{d \in {\mathcal {D}}_g} e^{-\frac{\mathrm{{dist}}(d,g)}{d_0}}, \end{aligned}$$

3 $$\begin{aligned} ps_g&= \sum _{d \in {\mathcal {D}}_g} s(d)e^{-\frac{\mathrm{{dist}}(d,g)}{d_0}}. \end{aligned}$$

The genomic distance of *d* to a specific gene *g* is denoted with *dist*(*d*, *g*). It is measured from the centre of the peak to the most $5'$-TSS of *g*. Using an exponential decay formulation proposed by Ouyang et al. [23], each peak is weighted by the distance to its linked gene. The parameter $d_0$ is controlling the effect of the decay and is set to 5000. The set ${\mathcal {D}}_g$ denotes the DHSs that are assigned to gene *g*. Details on the assignment are provided in the next section.

#### Window-based linkage

For each gene *g*, we consider a window *w* of size |*w*| centred at the most $5'$-TSS of *g*. We denote all DNase peaks *d*, and the ChromHMM regions *m* that overlap *w* with ${\mathcal {D}}_{g,w}$ and ${\mathcal {M}}_{g,w}$, respectively. Thus, we set $\mathcal D_g={\mathcal {D}}_{g,w}$ in Eqs. 1–3 to compute scores based on DNase1-seq data.

Additionally, we define an intersection operation $\cap _{H}$ between ${\mathcal {D}}_{g,w}$ and ${\mathcal {M}}_{g,w}$ such that only $d \in {\mathcal {D}}_{g,w}$ are retained that overlap by at least one 1*bp* with any $m \in {\mathcal {M}}_{g,w}$. Formally, that is:

4 $$\begin{aligned} {\mathcal {D}}_{g,w} \cap _{H} {\mathcal {M}}_{g,w}&= \{d| d \in D_{g,w}\ \wedge \ \exists m \in M_{g,w}:d \cap m \ne \emptyset \}, \end{aligned}$$

where $d \cap m$ indicates the overlap in genomic space of peak *d* and segment *m*.

We apply the $\cap _{H}$ intersection operation to ${\mathcal {D}}_{g,w}$ thereby obtaining ${\mathcal {D}}'_{g,w}$:

5 $$\begin{aligned} {\mathcal {D}}'_{g,w}&= {\mathcal {D}}_{g,w} \cap _{H} {\mathcal {M}}_{g,w}. \end{aligned}$$

Consequently, we use ${\mathcal {D}}_g={\mathcal {D}}'_{g,w}$ in Eqs. 1–3 and compute scores as described above. The window-based annotation is depicted in the upper part of Fig. 1.

#### Nearest gene linkage

In this linkage paradigm, a peak (*d*) or segment (*m*) is exclusively associated with its closest gene. Notably this implies that a peak or segment cannot be associated with more than one gene. Following this paradigm, we obtain ${\mathcal {D}}_{g,n}$, ${\mathcal {M}}_{g,n}$ and set ${\mathcal {D}}_g={\mathcal {D}}_{g,n}$ in Eqs. (1)–(3). As above, in Eqs. 4, 5, we intersect ${\mathcal {D}}_{g,n}$ with ${\mathcal {M}}_{g,n}$ using the $\cap _H$ operator and obtain ${\mathcal {D}}'_{g,n}$. The nearest gene annotation is visualized in the middle of Fig. 1.

#### HiC and HiChIP-based annotation

In addition to the window *w* centred at the TSS of gene *g*, we apply separate windows $v \in {\mathcal {V}}_{g}$ inferred from contacts of HiC or HiChIP experiments within one chromosome (Eqs. 6–8). The set ${\mathcal {V}}_{g}$ refers to all distant regions considered for gene *g*. We associate a chromatin contact to gene *g* if one of the two loop regions is located within a promoter search window of size *r*bp around the TSS of gene *g*. We refer to this search window as *loop window (LW)*. The set of all DHSs intersecting a window $v \in {\mathcal {V}}_{g}$ is denoted with ${\mathcal {D}}_{g,{\mathcal {V}}_g}$, and are included in the score computation. Because the chromatin conformation capture experiment suggests a direct interaction of a potentially far away region *v* with gene *g*, we do not apply an exponential decay to peak signals of that region. However, we did test whether applying the exponential decay in the distal regions would be beneficial for model performance and found that it is indeed not the case for both HiC and HiChIP experiments, since all features were shrunk towards zero (data not shown). Note that, in contrast to the promoter-centric window *w*, there might be more than one window *v* for a distinct gene *g*.

In addition to the promoter-centric features, we compute peak length $pl_g*$, peak count $pc_g*$, and peak signal $ps_g*$ in distal DHSs linked to gene *g* according to:

6 $$\begin{aligned} pl_g*&= \sum _{d \in {\mathcal {D}}_{g,{\mathcal {V}}_g}} l(d), \end{aligned}$$

7 $$\begin{aligned} pc_g*&= |{\mathcal {D}}_{g,{\mathcal {V}}_g}|, \end{aligned}$$

8 $$\begin{aligned} ps_g*&= \sum _{d \in {\mathcal {D}}_{g,{\mathcal {V}}_g}} s(d). \end{aligned}$$

The HiC/HiChIP-based annotation is explained in the bottom part of Fig. 1.

Finally, we integrate the ChromHMM information with the window-based annotation. To this end, we intersect ${\mathcal {D}}_{g,{\mathcal {V}}_g}$ with ${\mathcal {M}}_{g,V_g}$ and obtain ${\mathcal {D}}'_{g,{\mathcal {V}}_g}$ to reduce the number of regions associated with *g* from the distal regions $v \in {\mathcal {V}}_g$ (Additional file 1: Figure S1).

### Computation of TF-gene scores using $\textsc{TEPIC}$

In addition to the peak-based features $pl_g$, $pc_g$, $ps_g$, $pl_g*$, $pc_g*$, $ps_g*$, we estimate TF binding affinities using $\textsc{TEPIC}$ [24]. As introduced previously in Schmidt et al. [36], we compute TF affinities $a_{p,t}$ for TF *t* in peak *p* using $\textsc{TRAP}$ and aggregate the TF affinities to TF-gene scores $a_{g,t}$ according to

9 $$\begin{aligned} a_{g,t}&=\sum _{p \in {\mathcal {P}}_g} {\frac{a_{p,t}}{|p|-|m_t|+1}} \text { and} \end{aligned}$$

10 $$\begin{aligned} a_{g,t}&=\sum _{p \in {\mathcal {P}}_g} {\frac{a_{p,t}}{|p|-|m_t|+1}e^{-\frac{{\text{dist}}(p,g)}{d_0}}}, \end{aligned}$$

where ${\mathcal {P}}_g$ is the set of all DHSs assigned to gene *g*, reflecting the window-based, or nearest gene assignment. The variable |*p*| denotes the length of DHS *p*, $|m_t|$ denotes the length of the Position-Specific Energy Matrix (PSEM) $m_t$ representing the binding preference of TF *t*, dist(*p*, *g*) is the distance between peak *p* and gene *g,* and $d_0$ is a constant set to 5 kb [23]. Here, we used 726 PSEMs for *Homo sapiens*, obtained from $\text{JASPAR}$ [47], $\text{HOCOMOCO}$ [48] and the Kellis ENCODE motif database [49], which are included in the $\textsc{TEPIC}$ 2.0 repository [39].

As a baseline to be used in this study, we consider two promoter-centric windows to compute TF-gene scores: (1) TF-gene scores aggregating TF affinities in a promoter window of size 3 kb Eq. 9; (2) TF-gene scores aggregating TF affinities in an extended promoter window of size 50 kb including the exponential decay formulation of Eq. 10. To utilize the information offered by the chromatin conformation capture data, we additionally compute TF-gene scores $a_{g,t}*$ solely based on DHSs overlapping the LWs:

11 $$\begin{aligned} a_{g,t}*&=\sum _{p \in {\mathcal {D}}_{g,{\mathcal {V}}_g}} {\frac{a_{p,t}}{|p|-|m_t|+1}}. \end{aligned}$$

#### Generation of a null model for chromatin conformation data

To assess the significance of the included chromatin conformation data, we generated null models based on random sets of chromatin interactions that follow the distance distribution of the real data. Given a chromatin conformation capture data set ${\mathcal {C}}$ with $|{\mathcal {C}}|$ chromatin interactions and a distribution of distances of interacting sites $D({\mathcal {C}})$, we generate 10 random data sets ${\mathcal {R}}_i$, with $i \in [1,10]$ such that $|{\mathcal {C}}|=|{\mathcal {R}}_i|$, $\forall i$ and $D(R_i)=D({\mathcal {C}})$, $\forall i$. The random data sets are generated using the bedtools shuffle command [50]. Peak length $pl_g*$, peak count $pc_g*$, peak signal $ps_g*$, and TFBS prediction $a_{g,t}*$ values are computed for each random set $R_i$. In addition to the random chromatin interactions, we generated 10 random permutations of the gene annotation file to generate a base line for the extended feature space models (see below), also using bedtools shuffle. Random chromatin conformation data sets cannot be used to test the reliability of the extended feature space models, as all TF promoter features as well as the predicted TF binding sites that are associated to the loop site overlapping the loop window would not be affected by the randomization.

### Gene expression learning

Here, we briefly describe the machine learning techniques used in this study. An overview on the different feature setups is provided in Additional file 1: Figure S2, as well as in Table 1. The learning paradigm is sketched in Additional file 1: Figure S3.

#### Details on the linear model

Similar to a previous approach described in [24], we use linear regression with elastic net penalty implemented in the glmnet R-package [51] to predict gene expression. The elastic net combines two regularization terms, namely the Ridge (L2) and the Lasso (L1) penalty:

12 $$\begin{aligned} {\hat{\beta }}&=\underset{\beta }{\arg \,\min } ||y-X\beta ||^2 + \lambda [\alpha ||\beta ||^2 + (1-\alpha )||\beta ||]. \end{aligned}$$

Here, the feature coefficient vector is represented by $\beta$, the estimated coefficients are denoted by ${\hat{\beta }}$, *X* refers to the feature matrix, *y* refers to the response vector and the parameter $\lambda$ determines the total amount of shrinkage. Both the input matrix *X* and the response vector *y*, containing gene expression estimates, are log-transformed, with a pseudo-count of 1, centred and normalized. The parameter $\alpha$, which is optimized in a grid search from 0.0 to 1.0 with a step-size of 0.01, controls the trade-off between Ridge and Lasso penalty. Model performance is assessed on a hold-out test data set in a ten-fold outer Monte Carlo cross-validation procedure with $80\%$ of the data randomly chosen to form the training data and $20\%$ as test data. The $\lambda$ parameter is fitted in a six-fold inner cross-validation using the *cv.glmnet* function. We choose the $\lambda$ achieving the minimum cross-validated error, computed as the average mean squared error (MSE) on the inner folds *(lambda.min)*.

#### Details on the feature space

In this article, we build the feature matrix *X* in five different ways, listed in Table 1 and depicted in an exemplary manner in Additional file 1: Figure S2. As a baseline, we use our previously introduced promoter-centric models considering DHS-based features ($pl_g$, $pc_g$, $ps_g$) and TF-gene scores $a_{g,t}$. We refer to those as *Promoter: Peaks* and *Promoter: Peaks + TFs*, respectively.

Initially, we extended the promoter-based models only with peak-based features derived for loop sites ($pl_g*$, $pc_g*$, $ps_g*$), due to simplicity. We refer to the separate consideration of promoter and loop peak features as *Promoter + HiC/HiChIP: Peaks* and to the combined consideration as *Promoter + HiC/HiChIP: C Peaks*.

Finally, we construct a feature matrix that comprised all peak ($pl_g$, $pc_g$, $ps_g$,$pl_g*$, $pc_g*$, $ps_g*$) and all TF features ($a_{g,t}$, $a_{g,t}*$). We refer to this distinct consideration of peak features and TF-gene scores for promoter DHS and enhancer DHS as the *extended feature space (EF)*, as it expands the original feature space considerably and allows a more detailed interpretation of the models.

**Table 1:** Different combinations of features evaluated in this study

Name	Considered peak features	Considered TF features	Annotation
Promoter: peaks	Peak length $pl_g$		Window
	Peak count $pc_g$		Nearest gene
	Peak signal $ps_g$		ChromHMM
Promoter + HiC: peaks	Peak length $pl_g$		Window + HiC
Promoter + HiChIP: peaks	Peak count $pc_g$		Window + HiChIP
	Peak signal $ps_g$		ChromHMM
	Peak length $pl_g*$		
	Peak count $pc_g*$		
	Peak signal $ps_g*$		
Promoter + HiC: C peaks	Peak length $pl_g+pl_g*$		Window + HiC
Promoter + HiChIP: C peaks	Peak count $pc_g+pc_g*$		Window + HiChIP
	Peak signal $ps_g+ps_g*$		ChromHMM
Promoter: peaks + TFs	Peak length $pl_g$	Affinities in promoter DHS $a_{g,t}$	Window
	Peak count $pc_g$		ChromHMM
	Peak signal $ps_g$		
Promoter + HiChIP: peaks + TFs (EF)	Peak length $pl_g$	Affinities in promoter DHS $a_{g,t}$	Window + HiChIP
	Peak count $pc_g$	Affinities in loop DHS $a_{g,t}*$	ChromHMM
	Peak signal $ps_g$		
	Peak length $pl_g*$		
	Peak count $pc_g*$		
	Peak signal $ps_g*$		

#### Computation of p-values for TF features

To assess the significance of the features derived from the elastic net model in the extended feature space, we trained ordinary least squares (OLS) models for each cell line considering only TF and peak features that have been assigned a non-zero regression coefficient by the elastic net model. Using the F-test, a p-value is computed separately for each feature.

### Gene sets

For all models in this study, protein coding genes (gencode v19) are considered.

### Implementation changes in TEPIC

We have extended the $\textsc{TEPIC}$ framework by a novel module that allows the integration of any matrix describing genome-wide chromatin contacts. The new module requires two inputs. Firstly, it requires a file with paired intervals (e.g. HiC or HiChIP loops) to be included in the annotation and secondly a parameter specifying the size of the loop window *LW* should be provided by the user. The *LW* is the area around a gene that is being screened for a potential chromatin contact. Accessible regions overlapping with a chromatin contact are not subject to the exponential decay. Furthermore, regions overlapping the promoter window as well as the *LW* are not counted twice. They are only considered for the promoter window to avoid redundancy. Details on the formatting of the required input file and on the novel parameters are provided in Additional file 1.

### TF gene expression analysis

We generated a mapping of TF names to Ensemble GeneIDs using Biomart. To test whether TFs in a query set have a higher expression than expected, we sampled 1000 TF sets of the size of the query set from the entire TF universe (without replacement). Whether the difference in the expression distributions is significant or not is assessed with a Wilcoxon test.

### Network analysis

Protein–protein association (PPA) network analysis was conducted using the STRING database version 11 [52]. We obtained the PPA network for TFs found in the extended feature space analysis using four evidences from STRING (Experiments, Textmining, Databases, Co-occurrence). The final network was obtained using an interaction confidence score of 0.4 (default) and showing only proteins that are connected to at least one other protein (visualized in Fig. 6).

## Results

In this work, we developed an extension of our $\textsc{TEPIC}$ approach that aggregates regulatory events occurring in potentially distal regulatory sites to the gene level in a genome-wide fashion. Before we present the applicability and performance of this extension, we investigate common approaches that are widely applied in the community to establish PEIs ad hoc and use this comparison as a baseline for our novel methodology. Furthermore, we briefly describe differences in the HiC and HiChIP data used in this study.

### Local genomic architecture governs superiority of window or nearest gene-based approaches

**Fig. 2:**
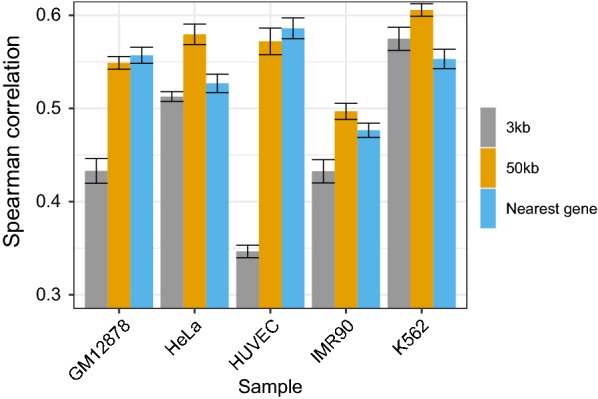
The performance of gene expression prediction models measured in terms of Spearman correlation on hold-out test data is shown for various models using peak length, peak count, and peak signal within the gene promoter regions. Two different window sizes ($3\,\text {kb}$, $50\,\text {kb}$) and the nearest gene approach are compared. We observe that the $50\,\text {kb}$ models outperform the $3\,\text {kb}$ models. Considering the $50\,\text {kb}$ models, there is a slight advantage of the window-based models over the nearest gene-based annotation

Previously, we have focused on window-based linkage approaches in $\textsc{TEPIC}$ [24, 36]. Here, we have taken a broader scope and included the nearest gene assignment as well. Just like the window-based approaches, this is another common strategy, used for instance by Gonzales et al. [25]. First we wanted to obtain a baseline, against which models considering chromatin conformation data can be compared later. We contrasted the performance of linear regression models predicting gene expression from the DNase1-seq derived features: (i) peak length, (ii) peak count and (iii) peak signal for window-based (${\mathcal {D}}_{g,w}$) and nearest gene linkage (${\mathcal {D}}_n$) for GM12878, HeLa, HUVEC, IMR90, and K562 cells. As shown in Fig. 2 and in Additional file 1: Figure S4a, $50\,\text {kb}$ windows outperform $3\,\text {kb}$ windows and compared to nearest gene approaches, the $50\,\text {kb}$ window leads to slightly better models for three out of five samples. In Additional file 1: Figure S4b the mean squared error (MSE) for 9000 randomly selected, individual genes is shown using the DNase1-seq model for HeLa. Contrasting gene-specific prediction errors allows us to illustrate by comprehensive case-examples the existence of genome architecture-specific advantages and disadvantages of the PEI linkage approaches.

For example, the MSE of *RPL7A(ENSG00000148303)* is nearly twice as high using the nearest gene than the window-based annotation. As shown in Additional file 1: Figure S5a there seems to be a bidirectional promoter for *RPL7A* and *MED22*. The model suggests that this cannot be adequately covered by the nearest gene approach. A different scenario is depicted in Additional file 1: Figure S5b for the gene *HINT1(ENSG00000169567)*. This gene is located in a gene-sparse region surrounded by several DHS peaks, which seem to add a large portion of noise in the nearest gene approach. In contrast to that, for the gene *APOA2(ENSG00000131096)*, the nearest gene approach leads to a better performance as it neglects, in contrast to the window-based model, several DHS sites that seem to be associated with *TOMM40L* instead of *APOA2* (Additional file 1: Figure S5c). These genes, specifically *RPL7A, HINT1,* and *APOA2*, are highlighted in Additional file 1: Figure S4b. Overall, these results suggest that neither the window based, nor the nearest gene annotation, generalize well across all genes. Still, the 50 kb window-based approach tends to perform slightly better on average.

Besides, we found that the minimum distance of a DHS to a gene is also cell-type specific and not homogeneous across different cell lines (Additional file 1: Figure S6).

### Including ChromHMM states has diverse effects on model performance

To understand whether the performance of the models could be improved by a stricter selection of potential regulatory regions, we used promoter/enhancer states predicted with ChromHMM in GM12878, HeLa, HUVEC, IMR90, and K562, thereby reducing the set of considered DHSs (${\mathcal {D}}'_{g,w}$ and ${\mathcal {D}}'_{g,n}$). In Additional file 1: Figure S7 the results for the window and nearest gene annotation are contrasted. In general, the intersection of regulatory segments with DHSs (${\mathcal {D}}'$) reduces model performance compared to the ${\mathcal {D}}$ models. Only in case of HeLa, the nearest gene model does not lose performance.

This reduction in performance suggests that also relevant DHSs are removed from consideration. To investigate this hypothesis, we compared the mean DHS confidence score of removed DHSs in the ${\mathcal {D}}'$ models to the retained DHS (Additional file 1: Figure S8a). Opposing this hypothesis, we found that the confidence score for the retained peaks is higher for both window sizes and the nearest gene approach. Note that in case of the nearest gene annotation, the ChromHMM intersection represents a genome-wide filtering. Also, a large portion of removed peaks are linked to *Quiescent/Low, Weak Repressed Polycomb,* and *Weak transcription* chromatin states (Additional file 1: Figure S8b), which does not suggest that the removed regions have a regulatory role. Additionally, we observe that the average length of the considered DHSs tends to be shorter in ${\mathcal {D}}'$ compared to ${\mathcal {D}}$ models (Additional file 1: Figure S9).

### HiC resolution impacts the association of genes to long-range chromatin interactions

**Fig. 3:**
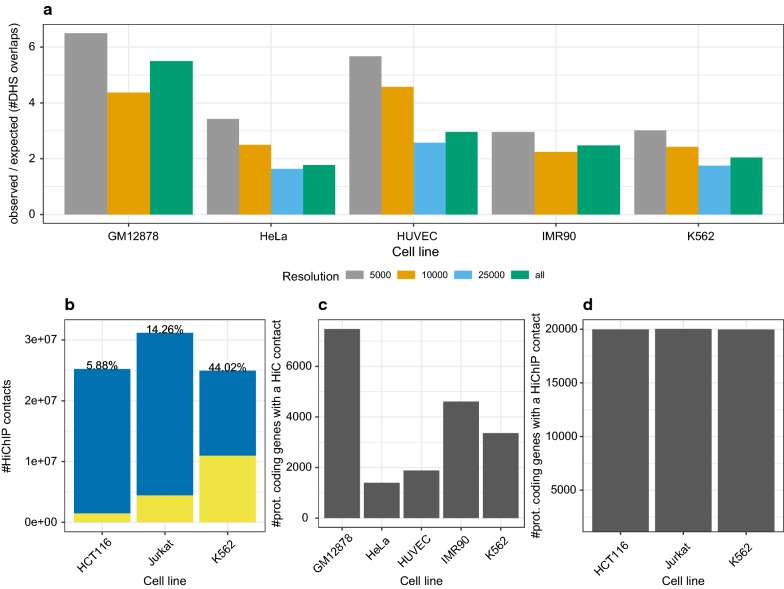
**a** The observed over expected ratio for the overlap between DHSs with HiC regions is shown for different cell lines and different HiC resolutions. **b** Percentage of HiChIP contacts overlapping with a DHS (yellow) compared to those not overlapping a DHS (blue). All HiChIP samples have a resolution of 5 kb. **c** The bar plot shows the number of prot. coding genes that overlap a HiC loop using a *LW* of 25 kb and a HiC resolution of 10 kb. **d** Analogously to **c** but with HiChIP data and a promoter search window of 5 kb. For **c** and **d** overlaps with DHSs are not considered

Before learning models using HiC or HiChIP data, we performed a few statistical analyses to better understand the characteristics of the chromatin conformation data. Compared to random regions, the real HiC data are enriched for DHS overlaps (Additional file 1: Figure S10). As expected, the enrichment reduces with a decreasing (numerically higher) HiC resolution (Fig. 3a). This analysis suggests that the choice of HiC resolution will affect any downstream analysis relying on DHS sites. As exemplarily shown in Fig. 3c for a *LW* of 25 kb and a HiC resolution of 10 kb, there are between 1000–7000 genes associated with a chromatin contact. In Additional file 1: Figure S11, we depict additional combinations for search windows and HiC resolutions. Generally, we observe that the number of genes associated with a loop reduces with a more precise, i.e. numerically smaller, HiC resolution. The *LW* used to link a HiC loop to a gene also influences the number of mapped genes. As expected, with an increasing search window size around the gene promoters, the number of genes that are linked to a loop is rising accordingly. Simultaneously the slope of the increase depends on the utilized HiC resolution. For example, as shown in Additional file 1: Figure S11, the increase in the number of genes is only marginal for the best resolution (5 kb), while it is more than three times as strong for the lowest one (25 kb). Compared to HiC data, it is striking how many genome-wide interactions are determined in HiCHIP data (Fig. 3b). For instance, in case of K562, there are $\approx 10{,}000{,}000$ chromatin interactions with a DHS in both loop sites, contrasted against $\approx 6000$ sites deduced from HiC (Additional file 1: Figure S11), considering a resolution of 5 kb for both data sets. As shown in Additional file 1: Figure S12a, there are still several magnitudes more HiChIP than HiC interactions, if a reduced HiChIP data set, filtered by q-Value or PET threshold, is considered.

We observe that almost all protein coding genes, following the hg19 reference annotation, are associated with a HiChIP contact (Fig. 3d). Upon a reduction of HiChIP contacts to those with a higher confidence, the number of affected genes stays above the levels of the HiC data (Additional file 1: Figure S12b). As one might expect, the mean distance between HiChIP sites of one chromatin contact is decreasing with a more stringent thresholding (Additional file 1: Figure S12c). Together with the count information from Additional file 1: Figure S12a, we note that the HiChIP data contains between 10,000 and 100,000 interactions within a distance of 5 kb–10 kb between the interacting sites.

### Effect of integrating HiC and HiChIP data on gene expression prediction

Due to the slight advantage of the window-based methodology, we decided to augment it using chromatin conformation capture data. Specifically, we attempt to replace the 50 kb models with 3 kb models that additionally consider DHSs linked to a distinct gene by chromatin conformation capture data. We hypothesize that the chromatin conformation capture data can provide more precise information about gene-specific regulatory events than the simplified 50 kb window, by considering loop information on a gene-specific level. To better understand the spatial characteristics of the data at hand, the resolution of the analysed data and the effect of different window sizes, we augment both the 3 kb and the 50 kb models with chromatin conformation data.

**Fig. 4:**
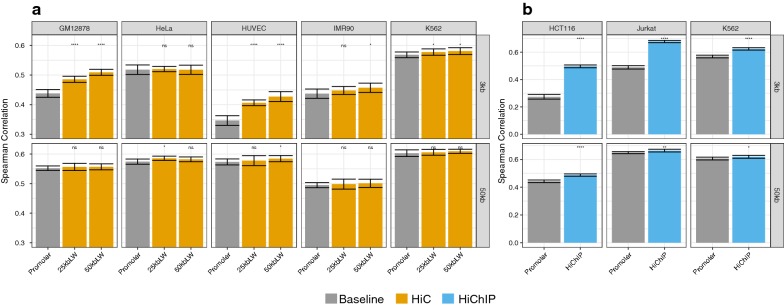
Model performance measured in Spearman correlation on hold-out test data considering chromatin contacts derived from **a** HiC data using two different search windows (25 kb and 50 kb) as well as **b** using HiChIP data. We considered two different promoter windows, a 3 kb and a 50 kb window. While for HiC, the 50 kb promoter windows led to better models than the 3 kb models, it is the other way around considering HiChIP data. Significance is assessed using a Wilcoxon test using the promoter model as the reference group ($p <0.0001$, **$p <0.01$, *$p <0.05$, ns: $p \ge 0.05$)

In addition to the promoter-centric models shown in Fig. 2, we trained linear models considering peak length, peak count, and peak signal of DHSs overlapping HiC and HiChIP loci, respectively. We refer to those features as *loop features*. Figure 4 illustrates that including HiC and HiChIP data can be beneficial for model performance. For HiC data, depicted in Fig. 4a, we observe a slight improvement in model performance, which is more pronounced in case of a 3 kb promoter window than with a 50 kb promoter window. However, for some of the cell lines the improvement is not significantly better than the promoter models.

As observed in our earlier studies [36], the 50 kb models outperform 3 kb models. Overall, our results indicate that a larger *LW* tends to be beneficial for model performance. This is especially pronounced for GM12878, HUVEC, and IMR90 cells using a 3 kb promoter window. This observation is likely to be directly linked to the dependency between the loop window size *LW* and the number of genes assigned with at least one HiC contact. Additionally, we assessed whether the gene expression models improve over the promoter models, due to the fact that more DHSs are included through looping regions. Therefore, we designed a control, by sampling random chromatin contacts of the same size as the real data (see “Materials and methods”). As shown in Additional file 1: Figure S13, the random models neither significantly improve over the promoter nor the real HiC data, supporting the potential biological relevance for cell lines GM12878, HUVEC, and IMR90 (see Fig. 4a).

In case of HiChIP data, illustrated in Fig. 4b, we see a stronger improvement of model performance upon inclusion of the loop features. Here, models extending the 3 kb promoter window perform at least as good, or better than those extending the 50 kb promoter window. It is possible, that the higher number of relatively short HiChIP interactions is responsible for this observation, which is in opposing to what we have seen with HiC data.

In addition, we analysed the performance of the HiChIP models in comparison to random interactions, similar as for HiC before. As shown in Additional file 1: Figure S14a, random models neither significantly improve over the promoter, nor the real HiChIP data, supporting the biological relevance of the HiChIP data as well. Moreover, as shown in Additional file 1: Figure S15, only a separate consideration of promoter and *loop features* leads to an improvement in model performance.

To better understand the relation between model performance and HiChIP interaction count, we reran the HiChIP models using different PET cut-offs. As depicted in Additional file 1: Figure S16a, model performance constantly drops with a stricter cut-off in $3\,\text {kb}$ promoter windows for HCT and Jurkat. In K562 the drop is less after a threshold of two, similar to what we observe for all samples using a $50\,\text {kb}$ promoter window. As expected, we notice that the number of putative HiChIP interactions that can be considered in the model, that is HiChIP contacts overlapping a DHS in both interacting loci, constantly drops with a more stringent cut-off (Additional file 1: Figure S16b). Taken together, these results suggest that several of the excluded interactions are already included in the $50\,\text {kb}$ promoter models, avoiding a performance drop. This is supported by the observation that the median distance of interacting sites is zero for larger PET thresholds (Additional file 1: Figure S16c). Secondly, it seems worthwhile to consider all suggested HiChIP interactions in the model without imposing a PET (or q-value) cut-off as all interactions are required to achieve the best model performance.

Compared to promoter-only models, filtering DHS with ChromHMM can improve models that are considering loop features (Additional file 1: Figure S17).

We chose the 3 kb HiChIP annotation for further examination in an extended feature space approach using TF affinities, because these models achieved the best performance with purely peak-based features. As described in the next section, we attempt to decipher the regulatory impact of TFs binding in promoters and enhancers suggested by the chromatin conformation capture data.

### Modelling of TF binding to distal regulatory elements

**Fig. 5:**
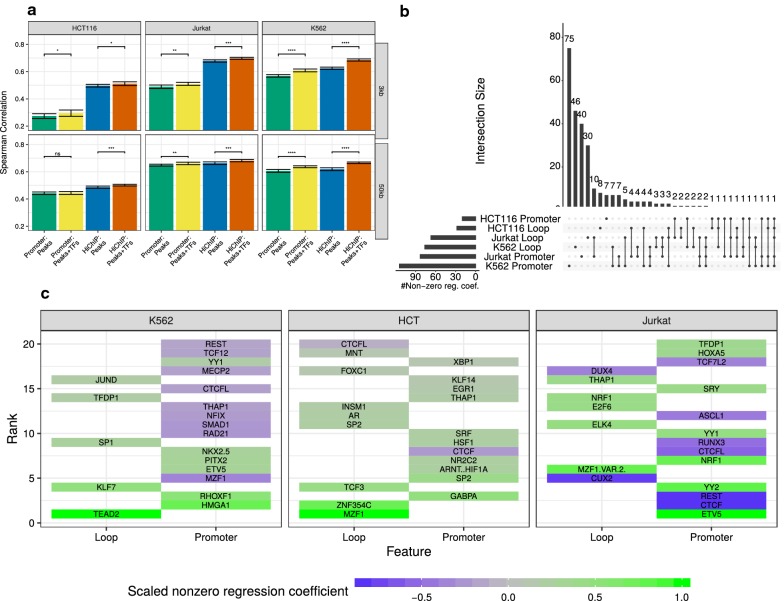
**a** Model performance assessed in terms of Spearman correlation on hold-out test data for models including TF-gene scores computed in the promoter and in the distal enhancers. Generally, including TF predictions improves model performance compared to considering only peak features. Significance is assessed using a Wilcoxon test using the promoter model as the reference group (****$p <0.0001$, **$p <0.01$, *$p <0.05$, ns : $p \ge 0.05$). **b** UpSet plot showing the relationship between TFs with a non-zero regression coefficients inferred by the extended feature space models. **c** Ranking of the top 20 TFs by their absolute regression coefficients for each cell line. The colour code indicates the mean scaled regression-coefficient of the TFs computed in a ten-fold outer cross-validation

In our earlier works, we showed that models including TF affinities can be used to learn about the tissue-specific regulatory activity of TFs [24, 36] and can be extended to investigate regulation of differential gene expression, for example in Durek et al. [37]. Therefore, we asked whether adding features for each TF would change the prediction performance. As shown in Fig. 5a, including TF affinities derived for DHSs around the promoter of genes, improves the performance of the linear models, compared to those models that are based solely on peak features.

Having the information about chromatin loops, we can not only consider TF affinities in the promoter, but also in distal sites determined with HiChIP data. We trained models using an extended feature space considering each TF separately for the promoter and aggregated over the distal loop windows, for the K562, HCT116, and the Jurkat cell line. The distinct inclusion of these features further improves model performance (Fig. 5a, Additional file 1: Figure S18), suggesting that we can gain additional insights on the role of TFs by examining their regression coefficients. As for the peak-based models (Additional file 1: Figure S14a), the random control experiments do not improve over the promoter nor the real HiChIP models (Additional file 1: Figure S14b). The UpSet plot in Fig. 5b depicts the overlap between TFs that have been assigned a non-zero mean regression coefficient in a ten-fold outer cross-validation procedure. The figure highlights that there are several factors occurring exclusively in promoter or loop regions, respectively.

Overall, we find the TFs ARNT::HIF1A, BHLHE41, CTCF, CTCFL, ETV5, ETV6, E2F8, GABPA, HINFP, HOXA5, INSM1, MEIS1, NKX2.8, NKX3.1, NRF1, REST, RUNX1, SRF, SRY, STAT1::STAT2, TCF7L2, TEAD2, TFDP1, THAP1, YY1, YY2, and ZNF384 to be selected as a feature in at least two of the three considered cell lines as a promoter feature. The TFs DUX4, E2F4, EGR4, GATA1::TAL1, GRHL1, KLF13, NRF1, NR2F1, PHOX2A, SOX9, SP2, YY1 are in at least two out of three cell lines as a loop feature.

Recalling that the HiChIP data were performed with an antibody targeting YY1 and the fact that YY1 binding sites are over represented in human core promoters [53], the prediction of YY1 as a common promoter feature is a validation of our computational approach. The appearance of YY2 may be due to the fact that the C-terminal binding domains of YY1 and YY2 are highly conserved. Indeed, ChIP-seq derived binding peaks of YY2 contained the YY1 motif at peak centres, indicating that YY2 binds similar regions and that there is an overlap of genes regulated by both TFs [54, 55].

Similar to YY1, also NRF1 has been shown to be essential for transcriptional regulation at the core promoter of several genes [56, 57]. The TF TFDP1 has been shown to bind to the promoter elements of genes that are related to the cell cyle [58]. Regarding the TFs commonly identified in loop regions, we find, for instance, that E2F4 has been suggested previously to bind to enhancer regions [59]. Binding sites of NR2F1 have been shown to coincide with high levels of the established enhancer marks P300 and H3K27ac [60].

**Fig. 6:**
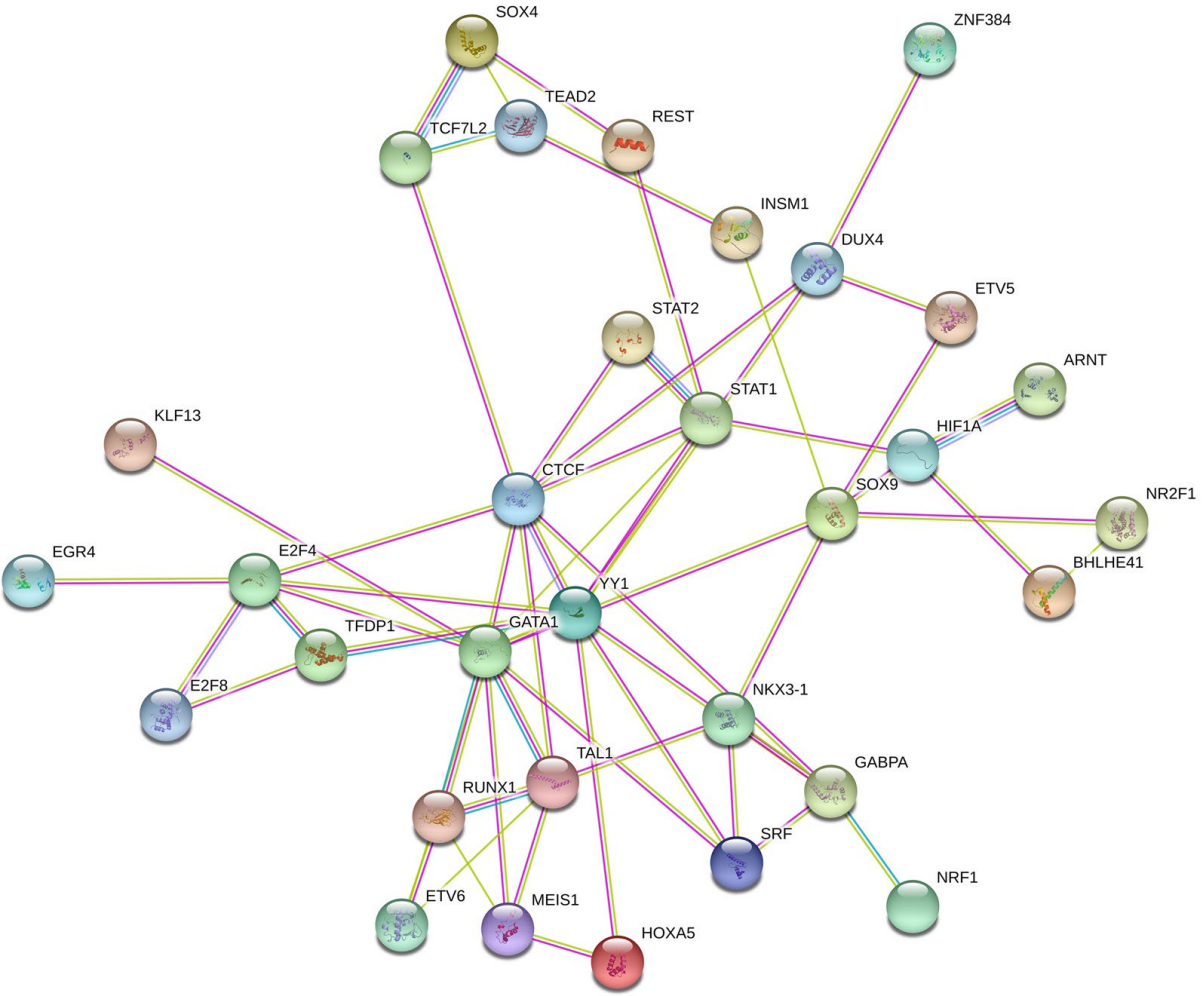
Protein–protein association network obtained from the $\textsc{STRING}$ database illustrating interactions among YY1 and TFs selected as a predictor in gene expression models for K562, Jurkat, and HCT116 in promoter and distal loop sites

Taking into account that the HiChIP data suggest a spatial proximity between the TFs bound to the promoter regions to those TFs bound to the loop regions, we investigated protein–protein associations using the $\textsc{STRING}$ database [52]. We selected the 40 TFs that occur in our models in at least two of the three cell lines, in either promoter or loop regions. The resulting network shows the 32 out of 40 TFs that are associated with at least one other TF according to $\text{STRING}$ (see Fig. 6 and “Materials and methods”). The analysis reveals that there are different modules among the selected TFs, where YY1 is placed in the centre, with associations to other hubs in the network, such as CTCF, STAT1, GATA1, and E2F4. The network suggests the formation of protein–protein complexes, or other types of protein associations, establishing links between enhancer and promoter regions.

In addition to this general analysis, we investigated the top 20 TFs ranked by their mean, absolute, regression coefficient across the ten-fold outer cross-validation, per cell line (Fig. 5c). For comparability and to simplify model interpretation, we scaled the regression coefficients by the maximum regression coefficient per sample.

For instance, HMGA1 is selected as a promoter feature in K562. This TF is known to act as an essential regulator for the mediator complex and the basal transcription machinery [61]. Another TF is YY1, which is among the top 20 TFs selected for K562 and Jurkat, supporting the validity of the ranking. On the loop sites, we find, for example JUND being among the top 20 factors in K562. This factor is known to support enhancer functions for instance in B cells and keratinocytes [62, 63]. MZF1, selected as a loop feature for both HCT116 and Jurkat, was previously associated with enhancer activity [64].

Interestingly, the knockdown of SP1 or KLF7, which are selected for K562 cells among the top loop features, has been shown to impact cellular differentiation and $\beta$-globin production, respectively [65]. While several TFs, e.g. HMGA1, a chromatin re-modeller which is known to be highly active in cancer [66], have a positive regression coefficient, indicating an activatory role of those TFs, we see that several TFs are assigned a negative regression coefficient. Our results are further supported by literature evidence, suggesting that ASCL1 [67], CTCF(L) [68, 69], CUX2 [70], MECP2 [71], MZF1 [64], NFIX [72], REST [73], TCF7L2 [74], and TCF12 [75] do carry out a repressive function.

The potential regulatory role of the TFs highlighted in Figs. 5c and 6 is additionally supported by the observation that the top 50 TFs with a non-zero regression coefficient per cell line have higher gene expression values than randomly sampled TFs (Additional file 1: Figure S19). To further improve the faith in the relevance of those highlighted TFs, we computed their statistical significance using the F-test incorporated in OLS models (“Materials and methods”, Additional file 1: Table S5). The OLS model is based on all features with a non-zero regression coefficient determined by the elastic net and, despite its simplifying assumption of feature independence, supports the majority of the tested TFs. Overall, our analysis of the extended feature space thus revealed a set of TFs that are likely frequently involved in enhancer-loop linkage involving YY1.

## Discussion

Associating regulatory regions to genes is still subject to ongoing research. In this work, we compared established methods to construct PEIs and present an extension of our $\textsc{TEPIC}$ approach to associate enhancers with genes using chromatin conformation capture data, exemplary using HiC and HiChIP data. We evaluated the different PEI linkage methods using predictive models of gene expression learned on DNase1-seq data.

Our results indicate that neither the widely used nearest-gene linkage nor the window-based PEI models are optimal. We illustrate that both approaches have distinct advantages and drawbacks. For example, in the nearest gene assignment, there is no common agreement, whether the TSS or transcription termination site (TTS) of a gene is used to calculate the genes’ distance to the putative enhancer. Also, in gene-dense regions, it is not obvious whether a peak should be uniquely assigned to only one or to multiple genes. Indeed, it was shown before that a distinct enhancer can influence the expression of various genes [76, 77]. On the other hand, the window-based linkage might generate many false-positive associations in gene-dense regions and likely misses distal enhancer regions, which in turn might be captured by the nearest gene approach in gene-sparse genomic loci. We have illustrated these points using multiple examples in Additional file 1: Figure S5. Notably, current research suggests that many enhancer–gene interactions are established only within TADs but only rarely across TAD borders [33]. This might argue in favour of window-based approaches and suggests to include TAD boundaries in nearest gene approaches to avoid assignments across TAD boundaries. It is of crucial importance for the field to understand the pros and cons of the assignment strategies, because they still form the basis for recent efforts trying to link enhancers to genes in multi-tissue scenarios [28]. Also, in settings were only few samples are available, a computational de novo assignment of regulatory regions to genes using correlation-based methods is not feasible. Approaches as proposed by Gonzales et al. [25], which iteratively reassign peaks to their presumable target gene, might also offer a remedy in such scenarios.

Our examination of the available HiC data suggests that the peak resolution has a strong impact on inferring PEIs. We showed that both the number of genes as well as the number of overlapping DHS sites largely depends on the HiC resolution. Notably, with a higher, i.e. numerically smaller, HiC resolution the number of genes associated with HiC loops almost remains constant with an increasing search window size. While models considering HiC data did improve in their ability to predict gene expression, the improvement was only marginal compared to the $50\,\text {kb}$ promoter model. This can be due to several reasons. One possibility might be that not all chromatin contacts are directly linked to transcriptional regulation and gene expression, as also suggested by Ray et al. [78]. For instance, loops could be part of higher-order structures and thereby indirectly influence cellular processes. This could be an explanation for the varying overlap of DHSs with HiC loops described in Fig. 5. It is likely that other methods, e.g. ChIA-PET, capture HiC [79], or HiChIP, which can enrich the sequencing libraries for distinct regions such as promoters of interest, lead to more precise contact maps in terms of both resolution and signal-to-noise ratio. Leveraging these more fine-grained technologies for gene expression modelling seems to be an opportunity to improve the prediction performance and to enhance our understanding on the underlying regulatory processes.

To examine this hypothesis, we learned gene expression prediction models using recently generated HiChIP data. In contrast to the HiC data, we see a stronger improvement in model performance. Importantly, the rather tight $3\,\text {kb}$ window focusing at the promoter augmented with HiChIP contacts, outperformed all $50\,\text {kb}$ model variants, suggesting that the interactions suggested by the HiChIP experiments are indeed meaningful and better than an average over all DHSs within $50\,\text {kb}$ of the TSS. As depicted in Fig. 4, the number of HiChIP contacts is several magnitudes higher than the number of HiC contacts. We noted that many of the high-quality HiChIP contacts belong to smaller range contacts, suggesting that HiChIP data also uncovers chromatin interactions between a gene’s TSS and intragenic enhancers. This might explain why the augmented $3\,\text {kb}$ models perform at least as good or better than the $50\,\text {kb}$ models as intragenic interactions are likely to be modelled in the HiChIP data. In this work we anchor our analysis on the promoter of genes for two main reasons: first, Promoters have been reported to be enriched for DHSs, e.g. compared to the gene-body or the TTS [80], suggesting that promoters are at the core of regulatory activity. Secondly, several HiC studies reported loop formation within a distinct gene, that is chromatin contacts between the TSS of gene *x* to intragenic positions of gene *x* up to its TTS, whereas loops from the TTS to other genomic locations have not been reported [81, 82].

A recent study by Furlong and co-workers might provide additional insights as to why the benefits of the chromatin interaction data, in particular HiC, showed limited improvement in performance. In Drosophila, it was shown that extensive rearrangements of chromatin in the three-dimensional space, which disrupted chromatin loops and changed TAD structures, did not lead to major gene expression changes [83]. These findings are coherent with our results, indicating that not all long-range chromatin interactions may be involved in the direct regulation of gene expression.

In contrast to the promoter-only models, including ChromHMM state segmentations did improve the performance of models considering HiC or HiChIP data, suggesting that both techniques lead to the inclusion of less relevant DHSs.

Wrapping up all these aspects, we tried to further improve the HiChIP models by incorporating TF affinities and extending the feature space of the model. This allowed a prioritization of inferred regulators in a promoter and a (distal) enhancer-specific point of view. We have shown that models based on this feature design can be comprehensively interpreted and lead to biologically meaningful and reasonable results. It is important to keep in mind that the TF activity inferred by the linear model should be seen as a majority vote across all genes for one sample. Thus, factors that have been reported to have both activating and repressing functions, e.g. CTCF [68] will be assigned a coefficient that represents the major contribution of the TF to the entire regulatory system at hand. Furthermore, we note that the *p*-values inferred by the OLS model should be considered with caution as the correlation in the activity among different TFs is not adequately addressed. Therefore, the relevance of a feature might be underestimated. Nevertheless, the *p*-values can be seen as an additional layer of evidence for the importance of a TF.

To make our approach generally applicable for the research community and to scale-up with new experimental technologies, we designed $\textsc{TEPIC}$s chromatin conformation extension to be able to integrate PEIs derived from any chromatin conformation capture technology. We believe that this extension together with the extended feature space annotation will be helpful to elucidate regulatory processes at promoters and enhancers.

## Conclusion

Overall, our study provides an unbiased comparison of prevalent PEI linkage strategies and shows that neither the established window-based PEI linkage nor the nearest gene linkage perform optimal. Further, we show that HiC and HiChIP data can both be used to integrate genome-wide chromatin contacts into predictive gene expression models. Thereby, we can not only often improve model performance, but, using our extended feature space formulation, enable users to obtain detailed insights into the promoter and enhancer-specific activity of TFs across distinct cell-types and tissues.

## Supplementary information

**Additional file 1.** Additional tables and figures.

## Data Availability

The code used to generate the results in this work is available online: https://github.com/SchulzLab/TEPIC. Data identifiers are provided in Additional file 1: Tables S1, S3 and S4.
