# Supplementary material for: Integrative prediction of gene expression with chromatin accessibility and conformation data
Source: Epigenetics Chromatin. 2020 Feb 6;13:4. doi: 10.1186/s13072-020-0327-0 (PMC7003490; doi:10.1186/s13072-020-0327-0)
Supplement: Supplementary file 1 — Additional file 1. Additional tables and figures. [file 13072_2020_327_MOESM1_ESM.docx]

*Integrative prediction of gene expression with chromatin accessibility and conformation data*

| ENCODE accession number | Data Type |
| --- | --- |
| ENCFF000DYC | Quantified mRNA of K562 |
| ENCFF441RET | DNase -1 seq of K562 |
| ENCFF000CZF | Quantified mRNA of GM12878 |
| ENCFF000SKV | DNase -1 seq of GM12878 |
| ENCFF000DJU | Quantified mRNA of IMR90 |
| ENCFF000SOC | DNase-1 seq of IMR90 |
| ENCFF000DNW | Quantified mRNA of HeLa |
| ENCFF000SPR | DNase-1 seq of HeLa |
| ENCFF000DUQ | Quantified mRNA of HUVEC |
| ENCFF001DNS | DNase-1 seq of HUVEC |
| ENCFF673ODZ | Quantified mRNA of JURKAT |
| ENCFF164FDV, ENCFF813IXN | DNase1-seq of JURKAT |
| ENCSR000CWM | RNA-seq reads of HCT116 |
| ENCFF081DDV, ENCFF291HHS | DNase1-seq of HCT116 |
| ENCFF916QPX | ChromHMM states for K562 |
| ENCFF869GUF | ChromHMM states for GM12878 |
| ENCFF147PPH | ChromHMM states for IMR90 |
| ENCFF654HNG | ChromHMM states for HeLa |
| ENCFF3970PB | ChromHMM states for HUVEC |

**Table S1:** Identifiers of ENCODE RNA-seq, DNase-1-seq, TF-ChIP-seq data, and ChromHMM files.

| Cell line | Number of identified DHS |
| --- | --- |
| K562 | 951.681 |
| GM12878 | 77.863 |
| IMR90 | 487.220 |
| HeLa | 673.093 |
| HUVEC | 164.551 |
| HCT116 | 153.738 |
| JURKAT | 322.906 |

**Table S2:** Sample Identifiers and number of DHSs.

| Supplement Identifier | Cell-line | Available resolutions |
| --- | --- | --- |
| GSE63525_GM12878_primary_HiCCUPS_looplist.txt.gz | GM12878 | 10kb |
| GSE63525_HUVEC_HiCCUPS_looplist.txt.gz | HUVEC | 5kb, 10kb, 25kb |
| GSE63525_HeLa_HiCCUPS_looplist.txt.gz | HeLa | 5kb, 10kb, 25kb |
| GSE63525_IMR90_HiCCUPS_looplist.txt.gz | IMR90 | 5kb, 10kb |
| GSE63525_K562_HiCCUPS_looplist.txt.gz | K562 | 5kb, 10kb, 25kb |

**Table S3:** Overview on the HiC data used in this study. The original data was obtained from Gene Expression Omnibus using the accession number GSE63525.

| Supplement Identifier | Cell-line |
| --- | --- |
| GSE63525_GM12878_primary_HiCCUPS_looplist.txt.gz | GM12878 |
| GSE63525_HUVEC_HiCCUPS_looplist.txt.gz | HUVEC |
| GSE63525_HeLa_HiCCUPS_looplist.txt.gz | HeLa |

**Table S4:** Overview on the HiChIP data used in this study. The original data was obtained from Weihrauch et al.

| **K562** | | | **HCT** | | | **Jurkat** | | |
| --- | --- | --- | --- | --- | --- | --- | --- | --- |
| Promoter | REST | * | Loop | CTCFL | *** | Promoter | TFDP1 | . |
| Promoter | TCF12 | . | Loop | MNT | NS | Promoter | HOXA5 | * |
| Promoter | YY1 | ** | Promoter | XBP1 | * | Promoter | TCF7L2 | *** |
| Promoter | MECP2 | . | Loop | FOXC1 | * | Loop | DUX2 | ** |
| Loop | JUND | NS | Promoter | KLF14 | ** | Loop | THAP1 | NS |
| Promoter | CTCFL | . | Promoter | EGR1 | * | Promoter | SRY | * |
| Loop | TFDP1 | NS | Promoter | THAP1 | * | Loop | NRF1 | * |
| Promoter | THAP1 | * | Loop | INSM1 | ** | Loop | E2F6 | NS |
| Promoter | NFIX | NS | Loop | AR | ** | Promoter | ASCL1 | *** |
| Promoter | SMAD1 | *** | Loop | SP2 | NS | Loop | ELK4 | . |
| Loop | RAD21 | ** | Promoter | SRF | *** | Promoter | YY1 | *** |
| Promoter | SP1 | NS | Promoter | HSF1 | ** | Promoter | RUNX3 | *** |
| Promoter | NKX2.5 | *** | Promoter | CTCF | ** | Promoter | CTCFL | *** |
| Promoter | PITX2 | NS | Promoter | NR2C2 | *** | Promoter | NRF1 | *** |
| Promoter | ETV5 | * | Promoter | ARNT::HIF1A | NS | Loop | MZF1.VAR.2. | . |
| Promoter | MZF1 | NS | Promoter | SP2 | . | Loop | CUX2 | NS |
| Loop | KLF7 | . | Loop | TCF3 | NS | Promoter | YY2 | *** |
| Promoter | RHOXF1 | *** | Promoter | GABPA | * | Promoter | REST | *** |
| Promoter | HMGA1 | *** | Loop | ZNF354C | NS | Promoter | CTCFL | *** |
| Loop | TEAD2 | *** | Loop | MZF1 | NS | Promoter | ETV5 | *** |

**Table S5:** The table provides the significance for the top20 TF features shown in Figure 5c computed using an ordinary least squared model trained on all features that have been assigned to a non-zero regression coefficients by the elastic net model (ns: p ≥ 0.1, p < 0.1: ., p < 0.05: *, p < 0.01: **, p < 0.001: ***).


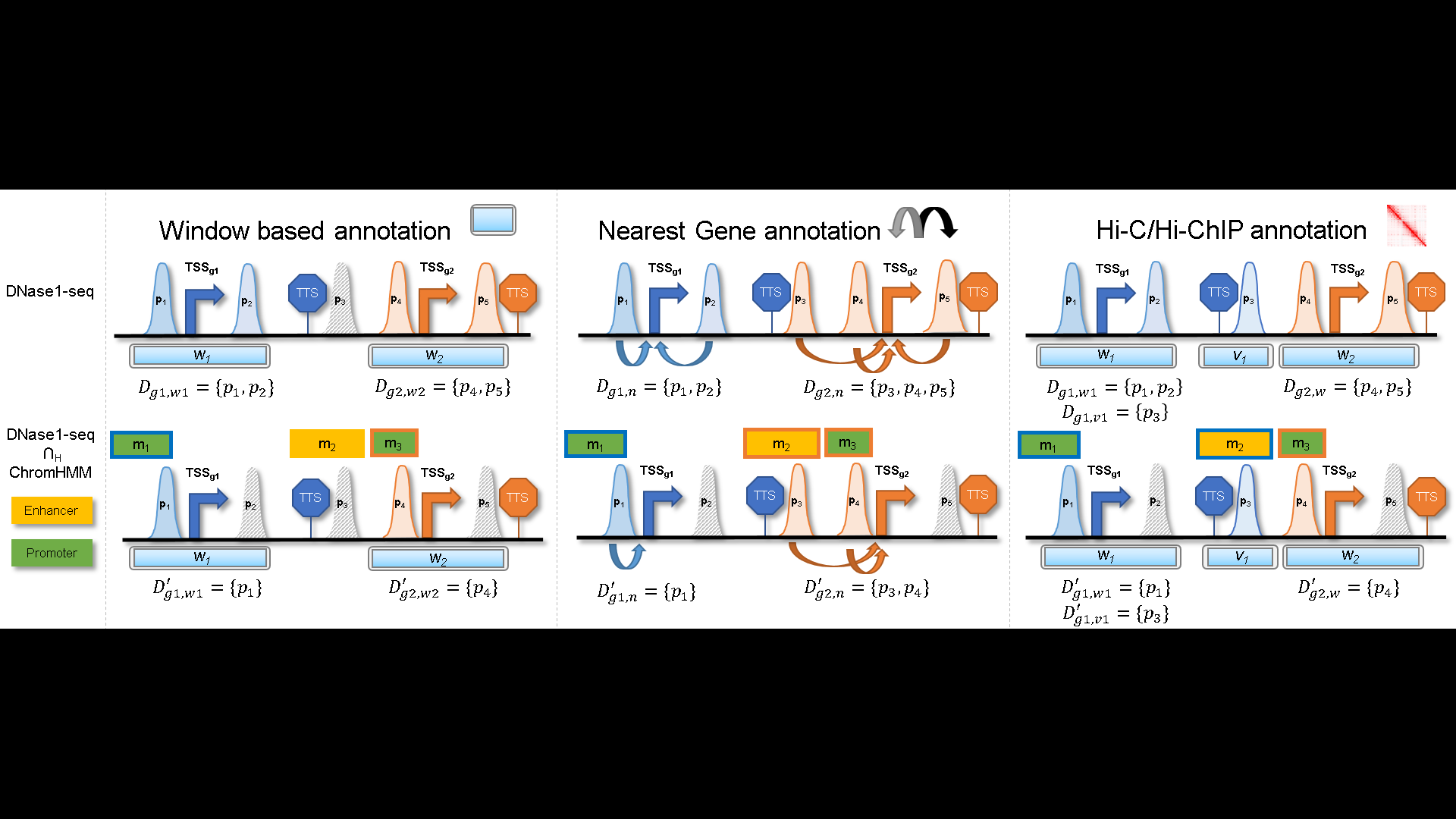


**Figure S1:** Here, the ChromHMM based filtering of the annotation versions shown in Figure 1 is shown. Briefly, only DHSs overlapping a ChromHMM promoter or enhancer segment are considered.

1. Promoter: Peaks

|  | Peak count | Peak length | Peak signal |
| --- | --- | --- | --- |
| Gene 1 | ${pc}_{1}$ | ${pl}_{1}$ | ${ps}_{1}$ |
| ... |  |  |  |
| Gene m | ${pc}_{m}$ | ${pl}_{m}$ | ${ps}_{m}$ |

1. Promoter + HiC/HiChIP: Peaks

|  | Peak count | Peak length | Peak signal | Peak count* | Peak length* | Peak signal* |
| --- | --- | --- | --- | --- | --- | --- |
| Gene 1 | ${pc}_{1}$ | ${pl}_{1}$ | ${ps}_{1}$ | ${{pc}_{1}}^{*}$ | ${{pl}_{1}}^{*}$ | ${{ps}_{1}}^{*}$ |
| ... |  |  |  |  |  |  |
| Gene m | ${pc}_{m}$ | ${pl}_{m}$ | ${ps}_{m}$ | ${{pc}_{m}}^{*}$ | ${{pl}_{m}}^{*}$ | ${{ps}_{m}}^{*}$ |

1. Promoter + HiC/HiChIP: C Peaks

|  | Peak count | Peak length | Peak signal |
| --- | --- | --- | --- |
| Gene 1 | ${pc}_{1}$*+* ${{pc}_{1}}^{*}$ | ${pl}_{1}$*+* ${{pl}_{1}}^{*}$ | ${ps}_{1}$ *+* ${{ps}_{1}}^{*}$ |
| ... |  |  |  |
| Gene m | ${pc}_{m}$*+* ${{pc}_{m}}^{*}$ | ${pl}_{m}$*+* ${{pl}_{m}}^{*}$ | ${ps}_{m}$ *+* ${{ps}_{m}}^{*}$ |

1. Promoter: Peaks + TFs

|  | Peak count | Peak length | Peak signal | Affinities TF t |
| --- | --- | --- | --- | --- |
| Gene 1 | ${pc}_{1}$ | ${pl}_{1}$ | ${ps}_{1}$ | $a_{1,t}$ |
| ... |  |  |  |  |
| Gene m | ${pc}_{m}$ | ${pl}_{m}$ | ${ps}_{m}$ | $a_{m,t}$ |

1. Promoter + HiChIP: EF Peaks + TFs

|  | Peak count | Peak length | Peak signal | Peak count* | Peak length* | Peak signal* | Affinities TF t | Affinities* TF t |
| --- | --- | --- | --- | --- | --- | --- | --- | --- |
| Gene 1 | ${pc}_{1}$ | ${pl}_{1}$ | ${ps}_{1}$ | ${{pc}_{1}}^{*}$ | ${{pl}_{1}}^{*}$ | ${{ps}_{1}}^{*}$ | $a_{1,t}$ | ${a_{1,t}}^{*}$ |
| ... |  |  |  |  |  |  |  |  |
| Gene m | ${pc}_{m}$ | ${pl}_{m}$ | ${ps}_{m}$ | ${{pc}_{m}}^{*}$ | ${{pl}_{m}}^{*}$ | ${{ps}_{m}}^{*}$ | $a_{m,t}$ | ${a_{m,t}}^{*}$ |

**Figure S2:** The different feature matrices used in this study are shown.


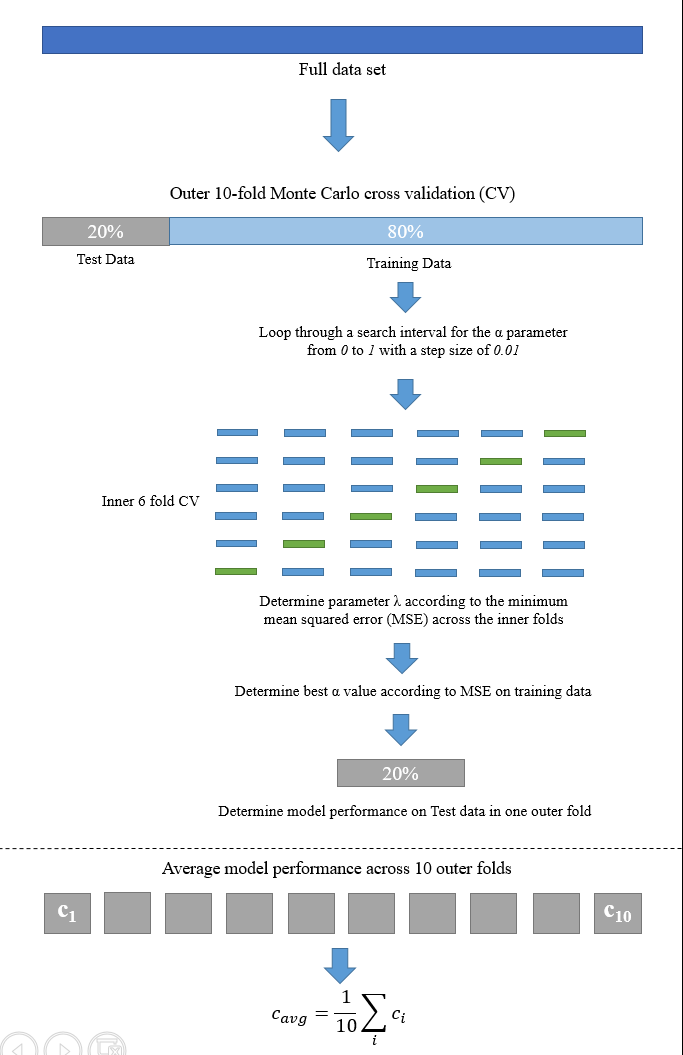


**Figure S3:** A scheme of the used linear regression paradigm.


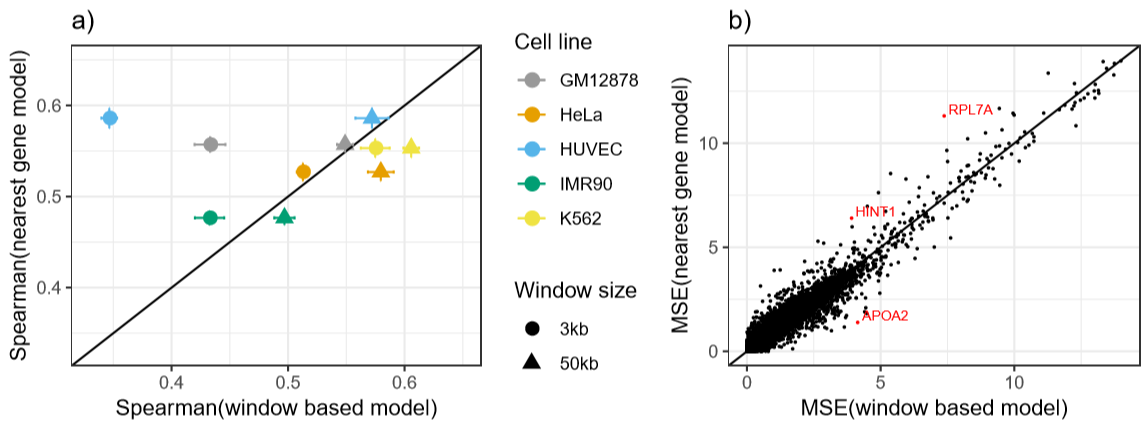


**Figure S4:** a) Spearman correlation achieved by regression models of gene expression using aggregated DNase1-seq data for a window based (x-axis) and nearest gene based (y-axis) enhancer linkage. On average, the window-based approaches are outperforming the nearest gene association. In b) the mean squared error (MSE) between predicted and measured gene expression for 9000 randomly selected HeLa genes is shown for both window based and nearest gene models. For genes highlighted in red, Sup. Fig. 5 shows IGV screen-shots illustrating the chromatin landscape around them.


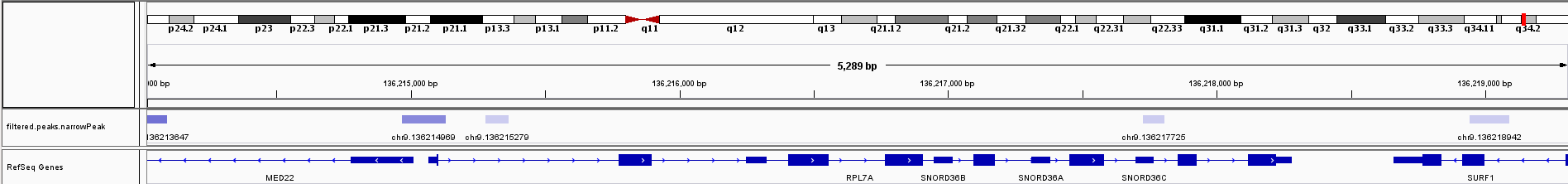
**a)**


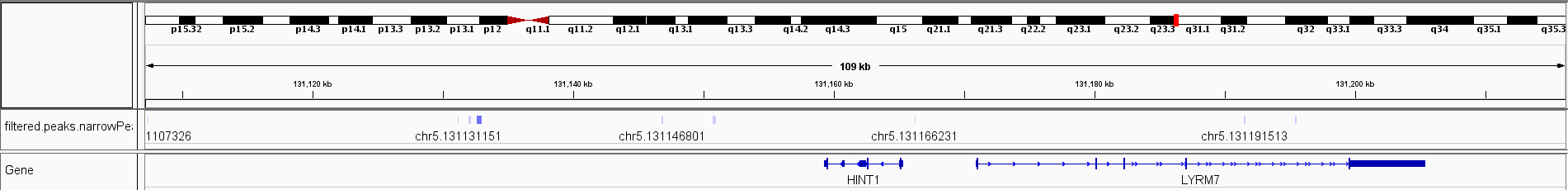
**b)**


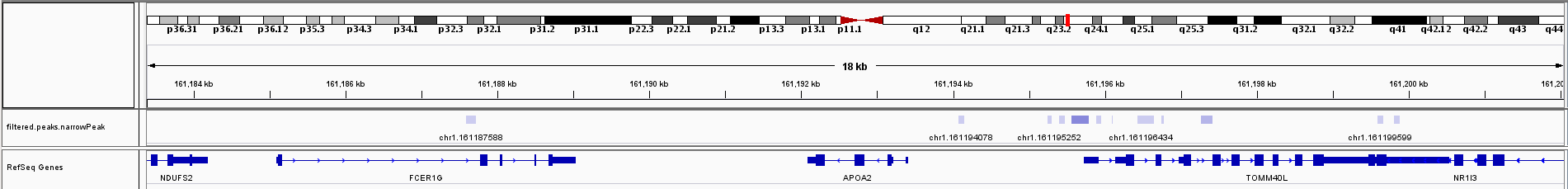
**c)**

**Figure S5:** IGV screenshots showing the JAMM peaks called in HeLa centered at three genes: a) RPL7A, b) HINT1 and c) APOA2.

**
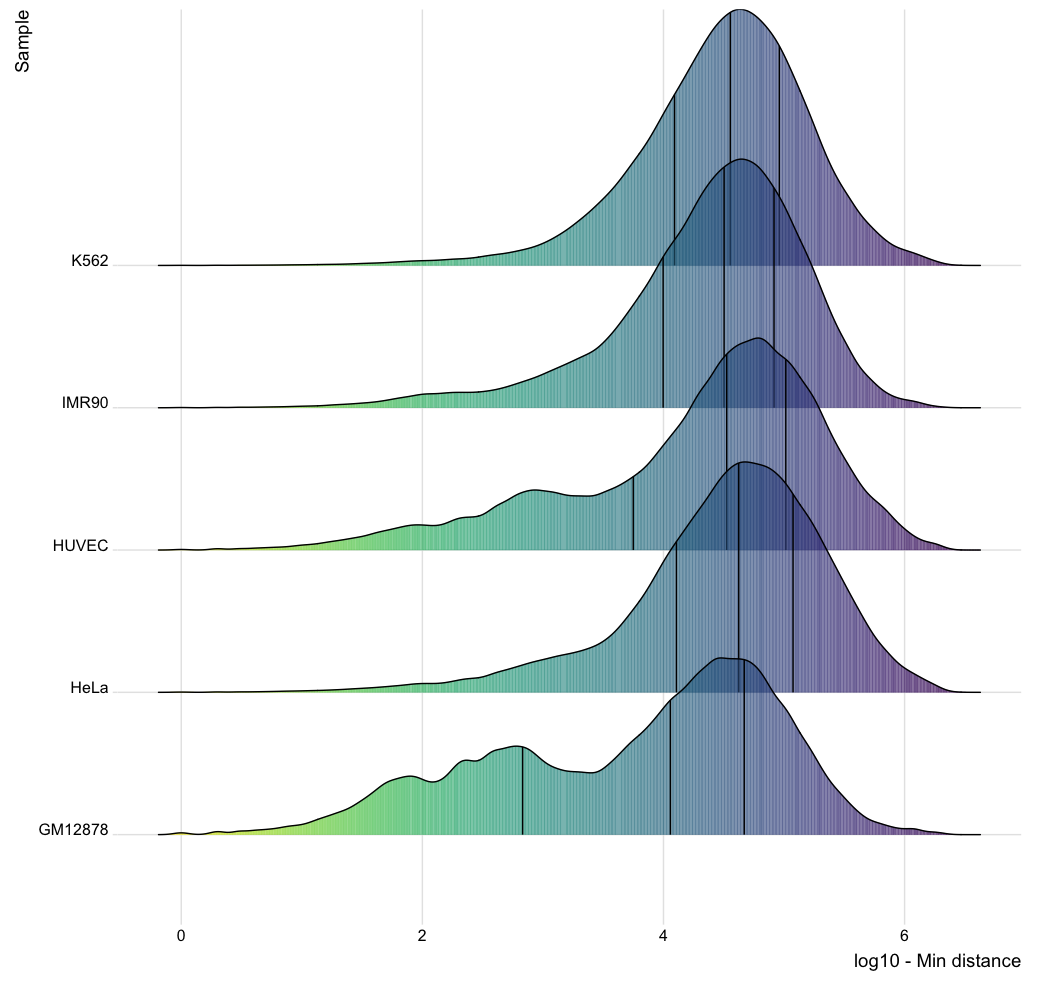
**

**Figure S6:** Here, the minimum distance (log scale) for each DHS to the nearest gene is shown for each considered cell-line. The distributions are similar for K562, IMR90 and HeLa, but different for HUVEC and GM12878.


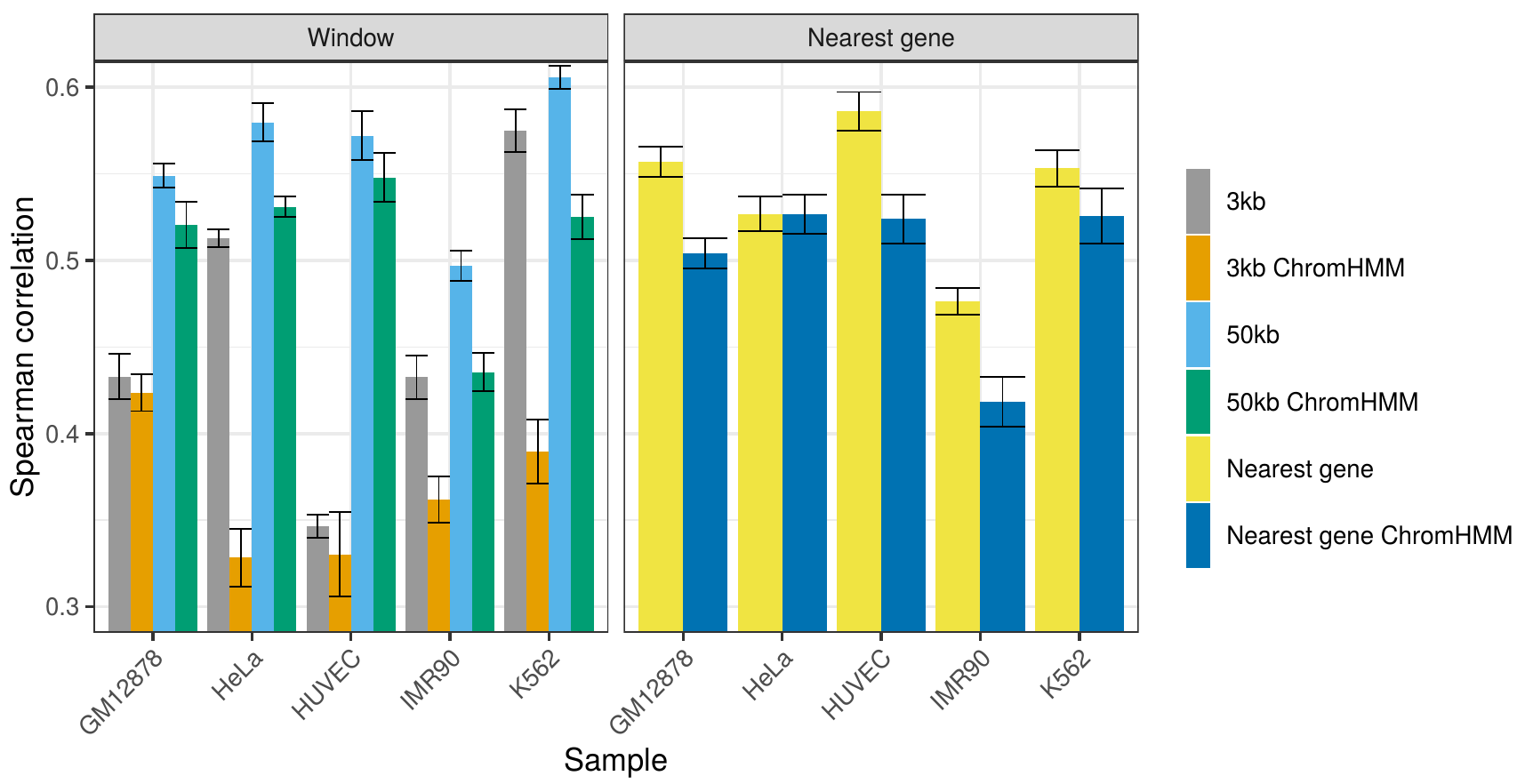


**Figure S7**: Here, model performance is shown in terms of Spearman correlation for Window and nearest gene based approaches with and without an additional filtering with ChromHMM.


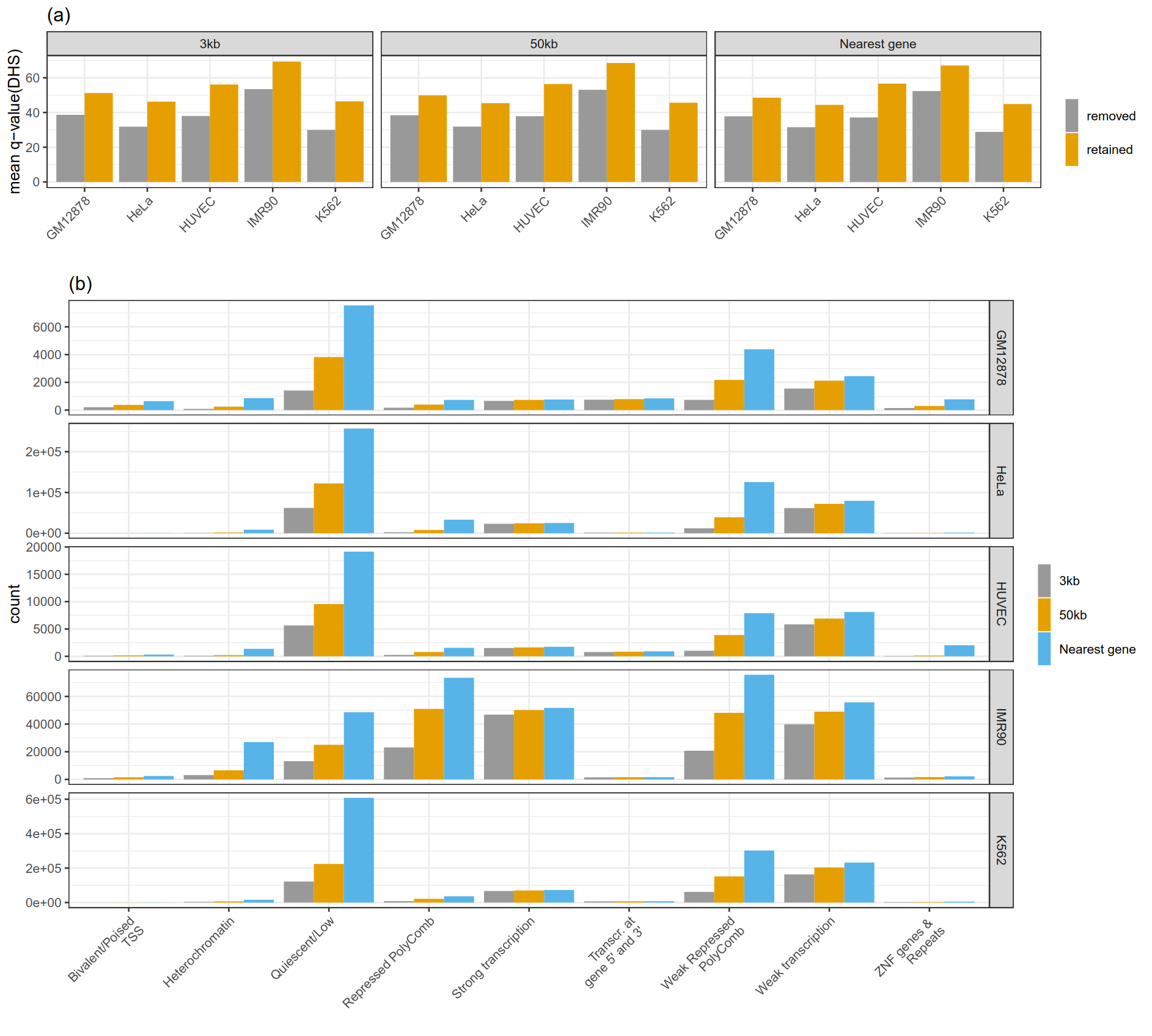


**Figure S8:** a) shows details on the characteristics of DNase1-seq peaks retained/removed by intersection with ChromHMM Promoter/Enhancer states. The y-axis shows mean values of –log (q-value), the confidence scores of JAMM. In part (a) the score of retained/removed peaks is shown, in (b) the count of removed peaks is shown per overlapping chromatin state.


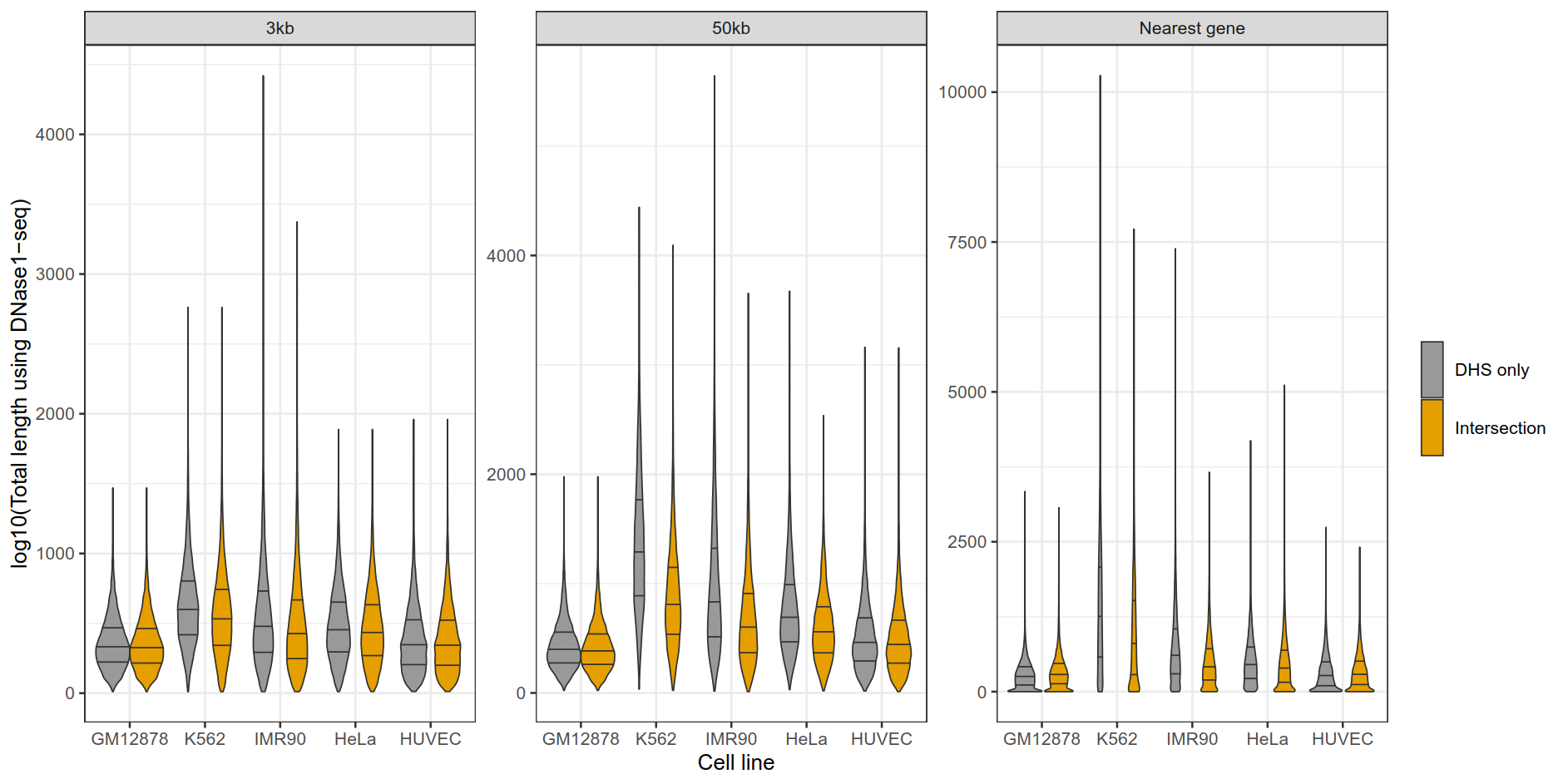


**Figure S9:** log10 of the average length of considered segments using DNase1-seq (a) and TF ChIP-seq data (b) using the window based linkage with two different window sizes (3kb, 50kb). The total length of associations based on DHS only are colored in grey, while orange represents DHSs intersecting ChromHMM segments.


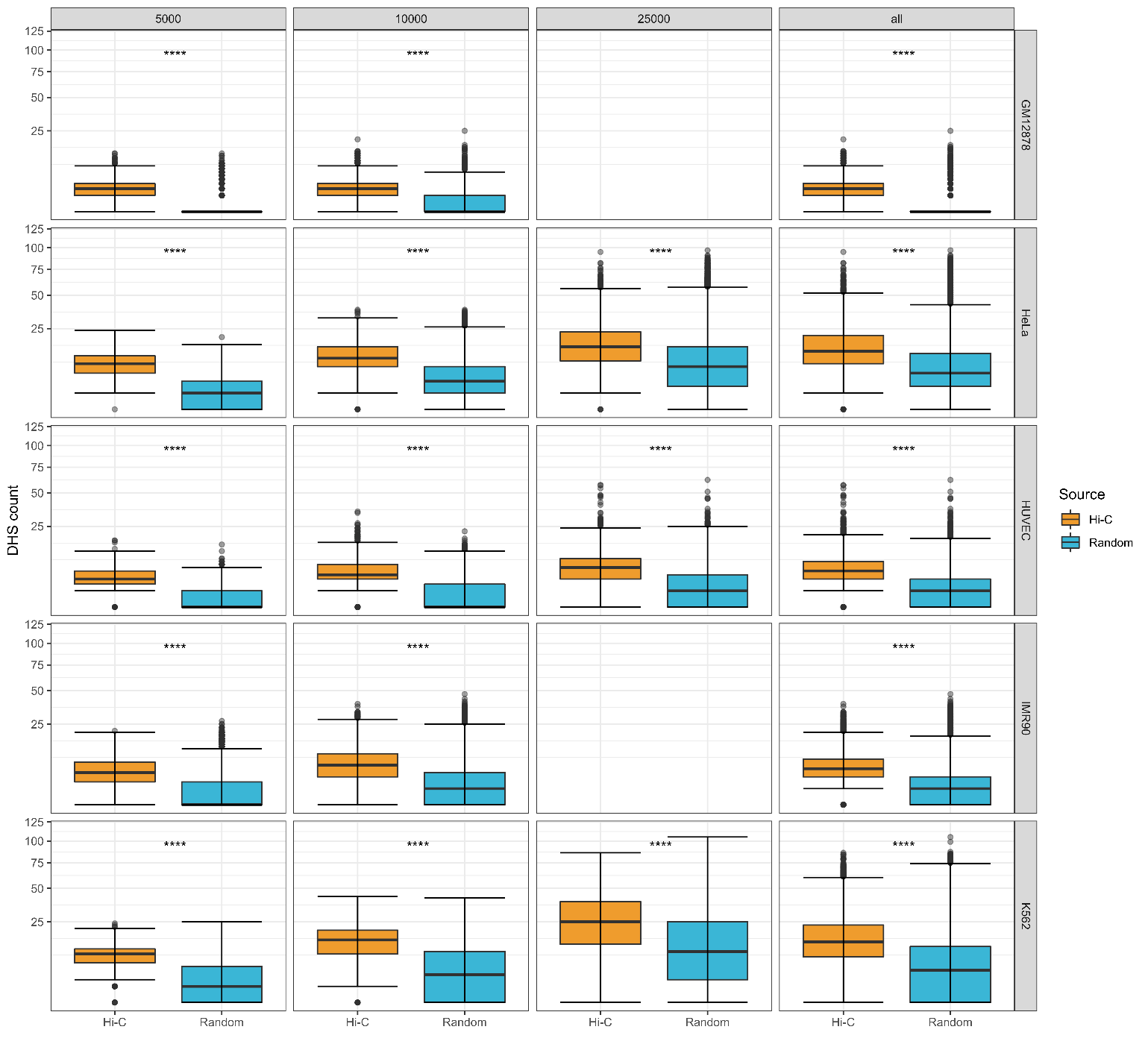


**Figure S10:** Assessment of the significance of DHS overlaps with HiC loops for different cell lines and different HiC resolutions (ns: p ≥ 0.05, p < 0.05: *, p < 0.01: **, p < 0.001: ***, p < 0.0001: ****).


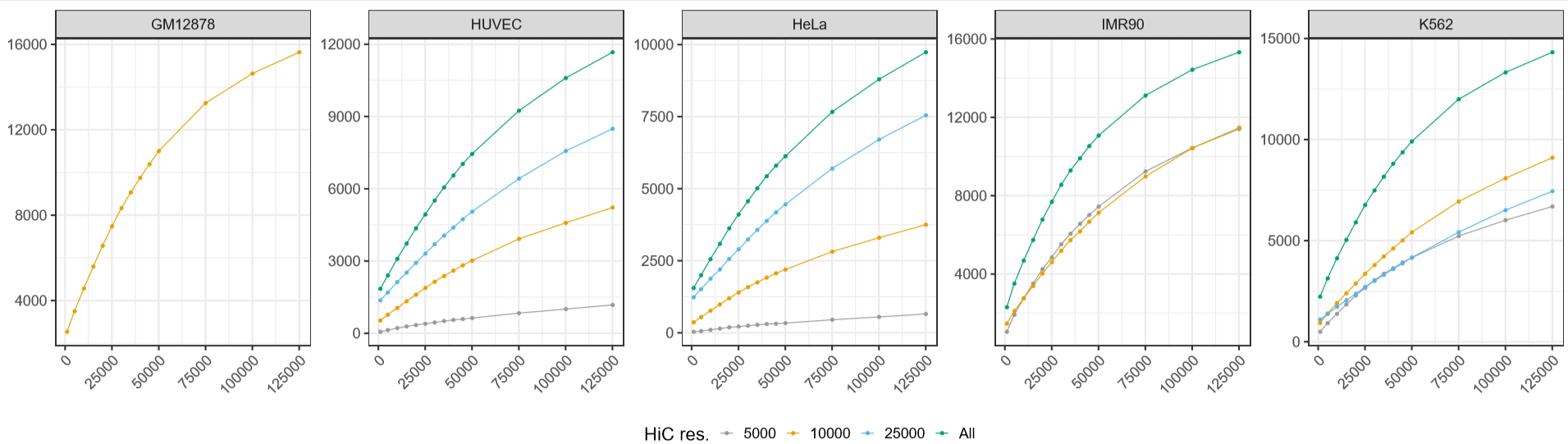


**Figure S11:** Here, the relationship between the number of genes (y-axis) overlapping a HiC loop to different HiC resolutions and various loop window sizes (x-axis) is depicted. Here, an additional overlap with DHSs is not required.


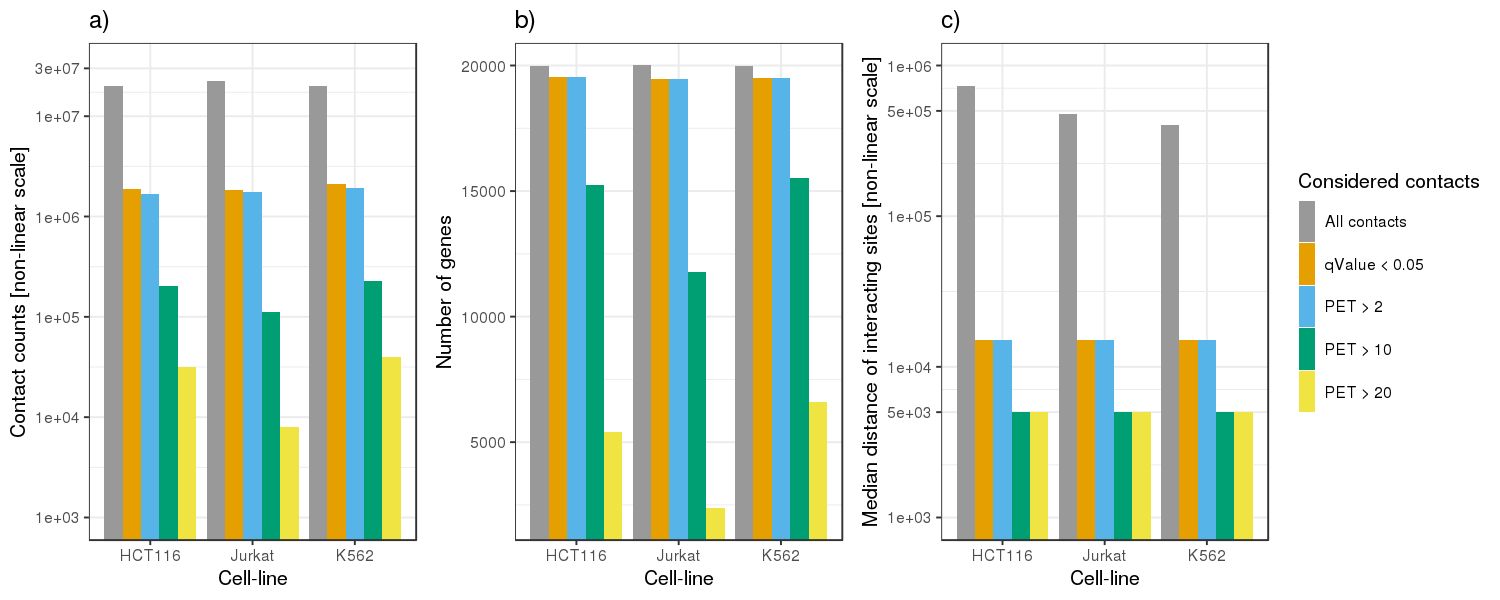


**Figure S12:** Filtering of HiChIP data and its influence on (a) contact counts, (b) gene counts and (c) the average distance of the interacting sites. Note that the data shown here relates to the raw HiChIP data. An overlap with DHSs is not considered (see Sup. Fig. 16b and c for that).


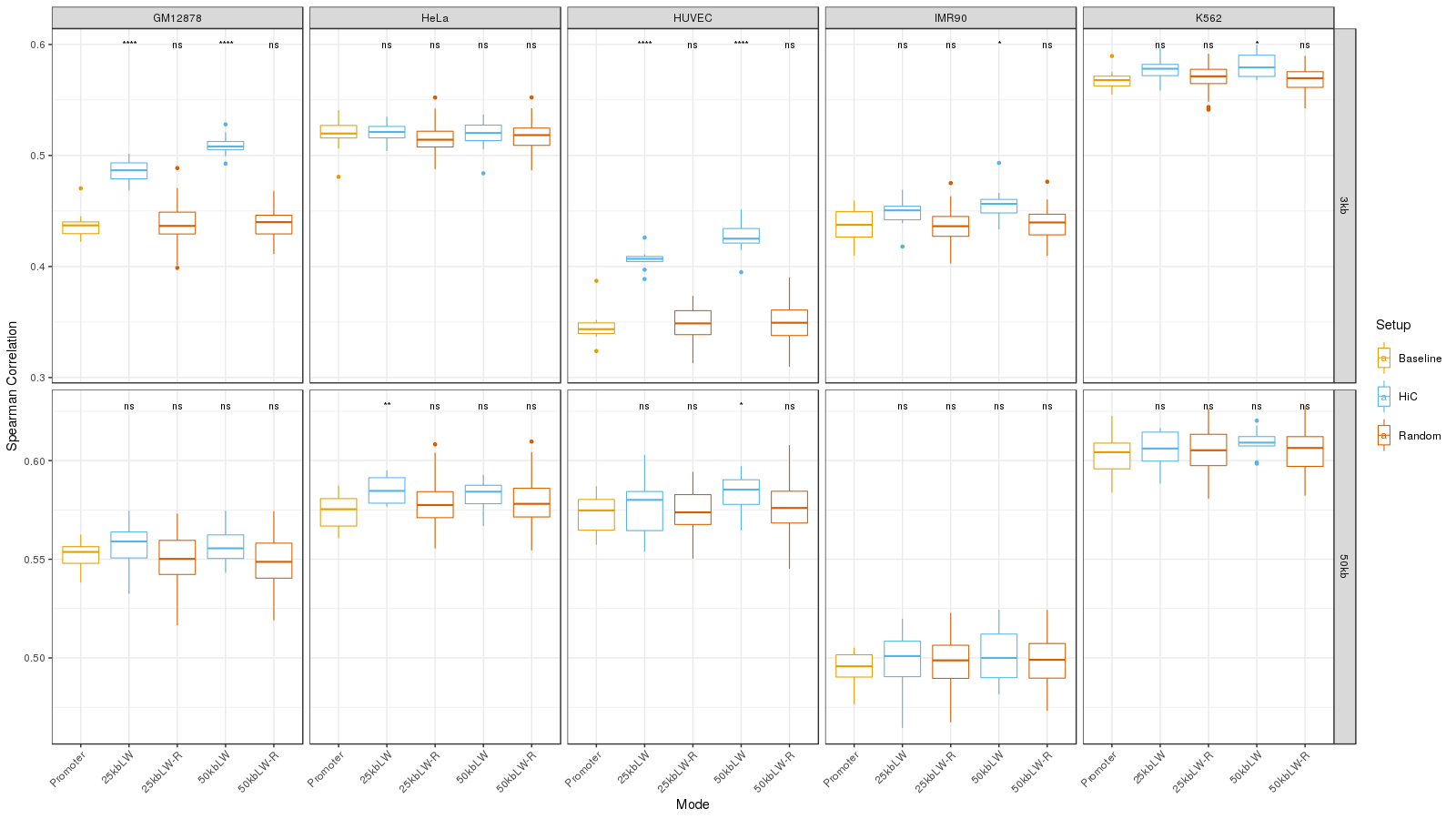


**Figure S13:** Here, we contrast the performance of gene-expression prediction models for GM12878, HeLa, HUVEC, IMR90 and K562 between Promoter (yellow), HiC (blue) and random HiC (orange) models with respect to two promoter windows: 3kb and 50kb. Using a Wilcoxon test, the statistical significance of the HiC and random HiC models compared to the sample specific promoter model is tested. Adding random interactions does not improve over the promoter models, while adding chromatin interactions derived from HiC data does improve models in several instances (ns: p ≥ 0.05, p < 0.05: *, p < 0.01: **, p < 0.001: ***, p < 0.0001: ****).


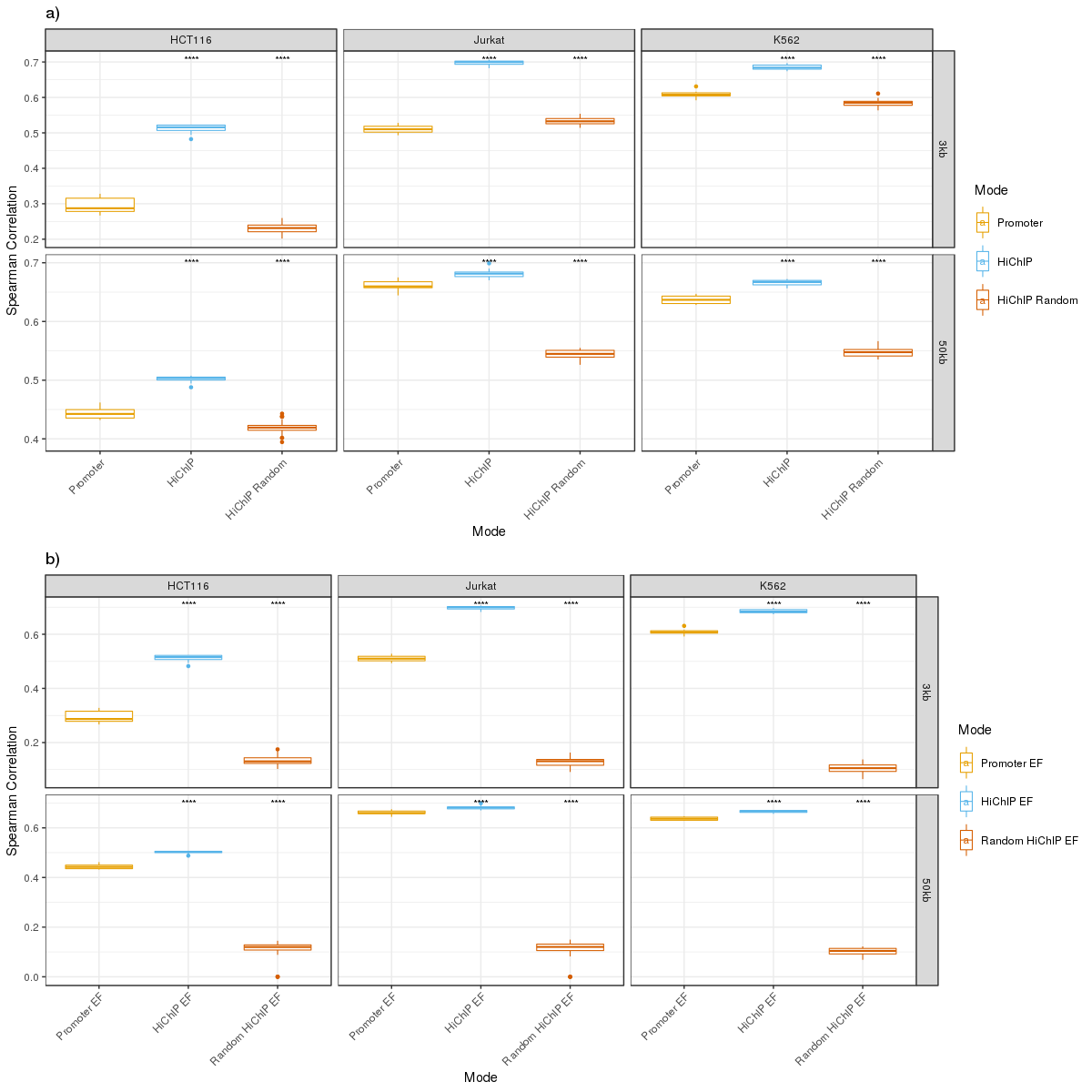


**Figure S14:**  a) Here, we contrast the performance of gene-expression predictions models for HCT116, K562 and Jurkat between Promoter (yellow), HiChIP (blue) and random HiChIP (orange) models with respect to two promoter windows: 3kb and 50kb. Using a Wilcoxon test, the statistical significance of the HiChIP and random HiChIP models compared to the sample specific promoter model is tested. Adding chromatin interactions derived from HiChIP data improves over the promoter based models in all tested conditions (ns: p ≥ 0.05, p < 0.05: *, p < 0.01: **, p < 0.001: ***, p < 0.0001: ****). b) Analogous to a) but based on the extended feature space which uses a gene-based randomization.


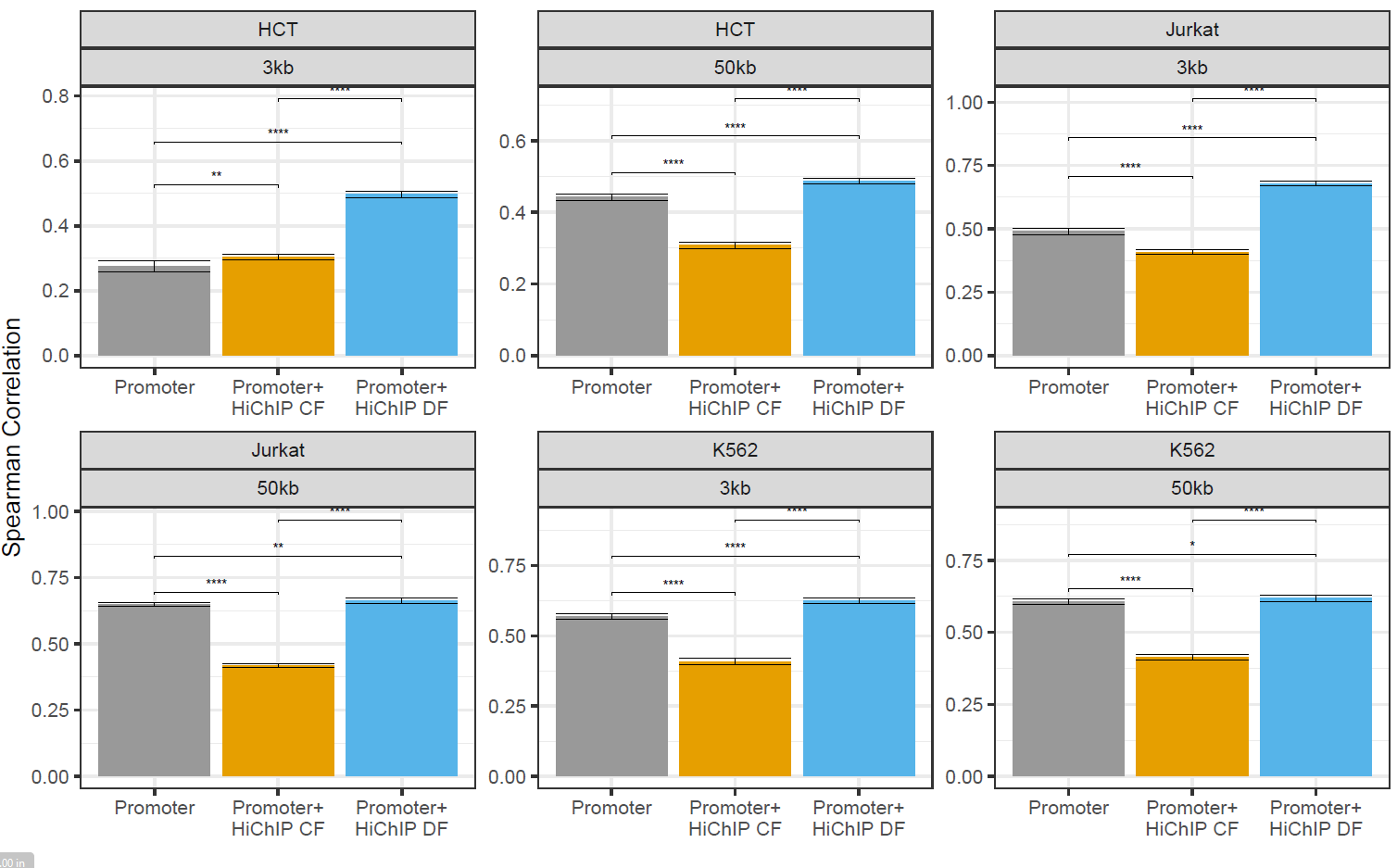


**Figure S15:** Here, the performance of spearman correlation obtained for gene-expression prediction models is shown for three different peak feature setups: Promoter only, Promoter and HiChIP peak features combined (CF), Promoter and HiChIP peak features considered separately (DF). Further, we considered two promoter window sizes: 3kb and 50kb (ns: p ≥ 0.05, p < 0.05: *, p < 0.01: **, p < 0.001: ***, p < 0.0001: ****).


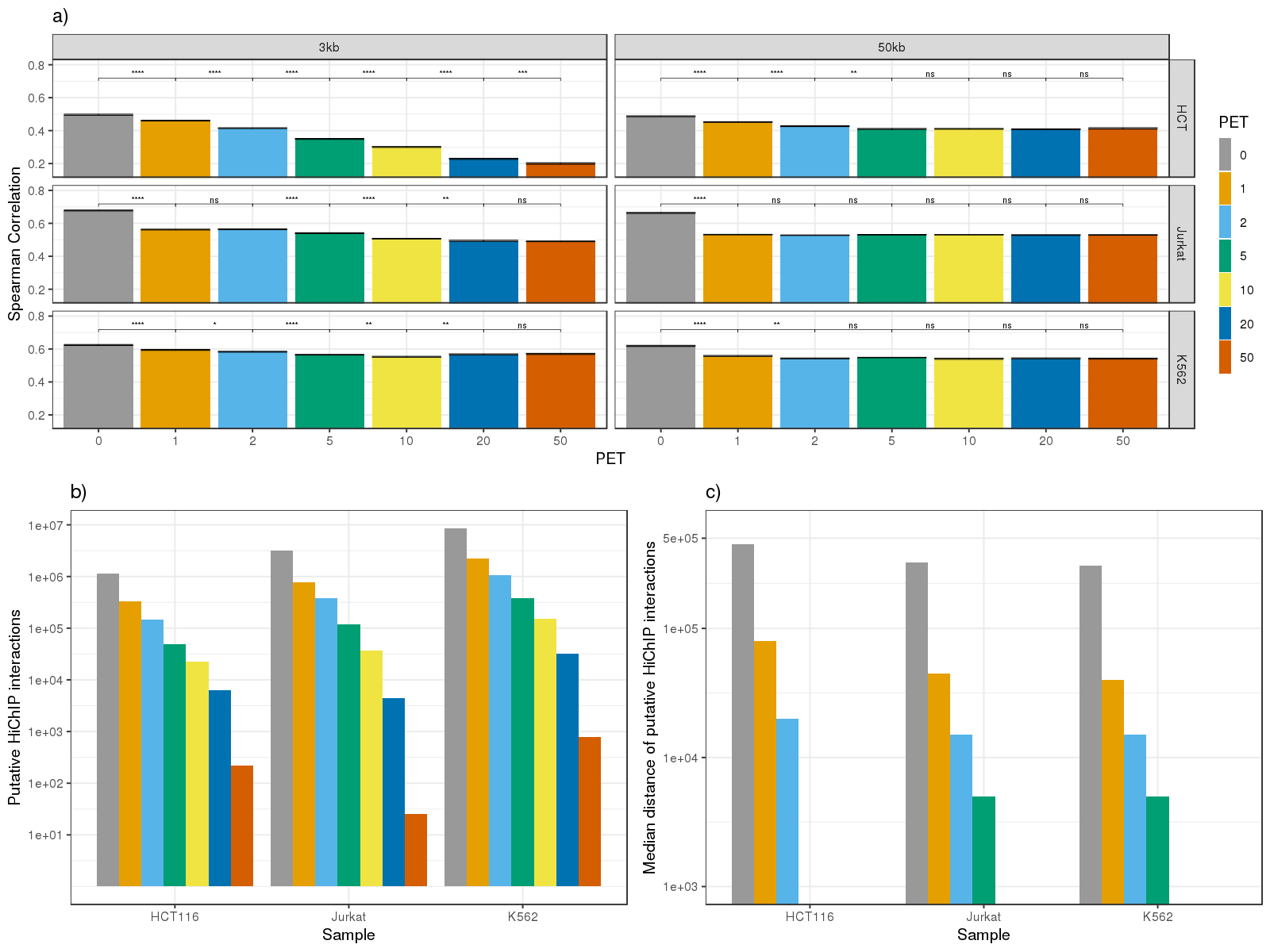


**Figure S16:** a) Here, the performance of spearman correlation obtained for gene-expression prediction models is shown for different HiChIP subsets with respect to different PET thresholds. More stringent cut-offs lead to worsen model performance. b) Thresholding the HiChIP data reduces the number of putative interactions that can be included in the model. A HiChIP intersection is putative if it can be included in the model, that is it overlaps a DHS in both interacting sites. As indicated in the Figure, the number of HiChIP sites that could be considered reduces drastically with an increasing cut-off. c) Thresholding the HiChIP counts by PET value reduces the median distance of the interacting sites. Interestingly, for a cut off of 5 (HCT116) and 10 (Jurkat and K562), respectively, the median distance is zero indicating that most of the putative high confidence interactions are direct neighbors in genomic space (ns: p ≥ 0.05, p < 0.05: *, p < 0.01: **, p < 0.001: ***, p < 0.0001: ****).


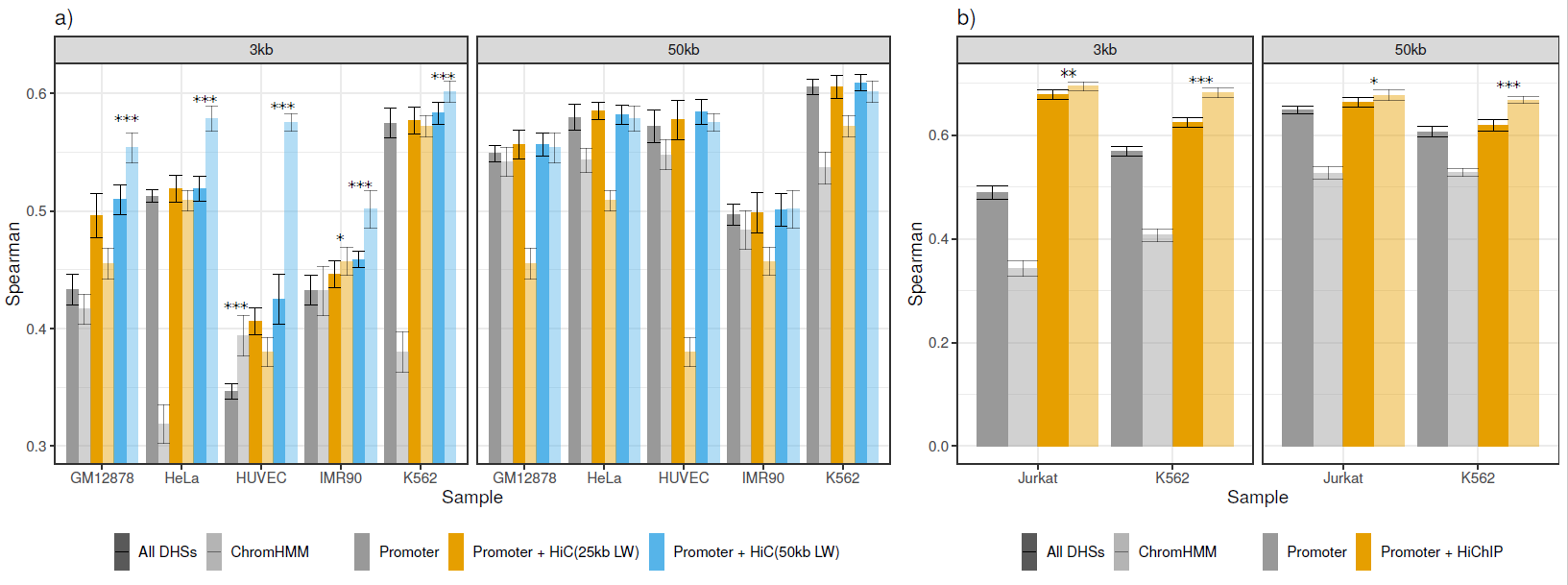


**Figure S17:** This figure illustrates the effect of a ChromHMM overlap with a) HiC and b) HiChIP regions on model performance. For HiC and HiChIP models, we consider only the DF feature representation (ns: p ≥ 0.05, p < 0.05: *, p < 0.01: **, p < 0.001: ***).


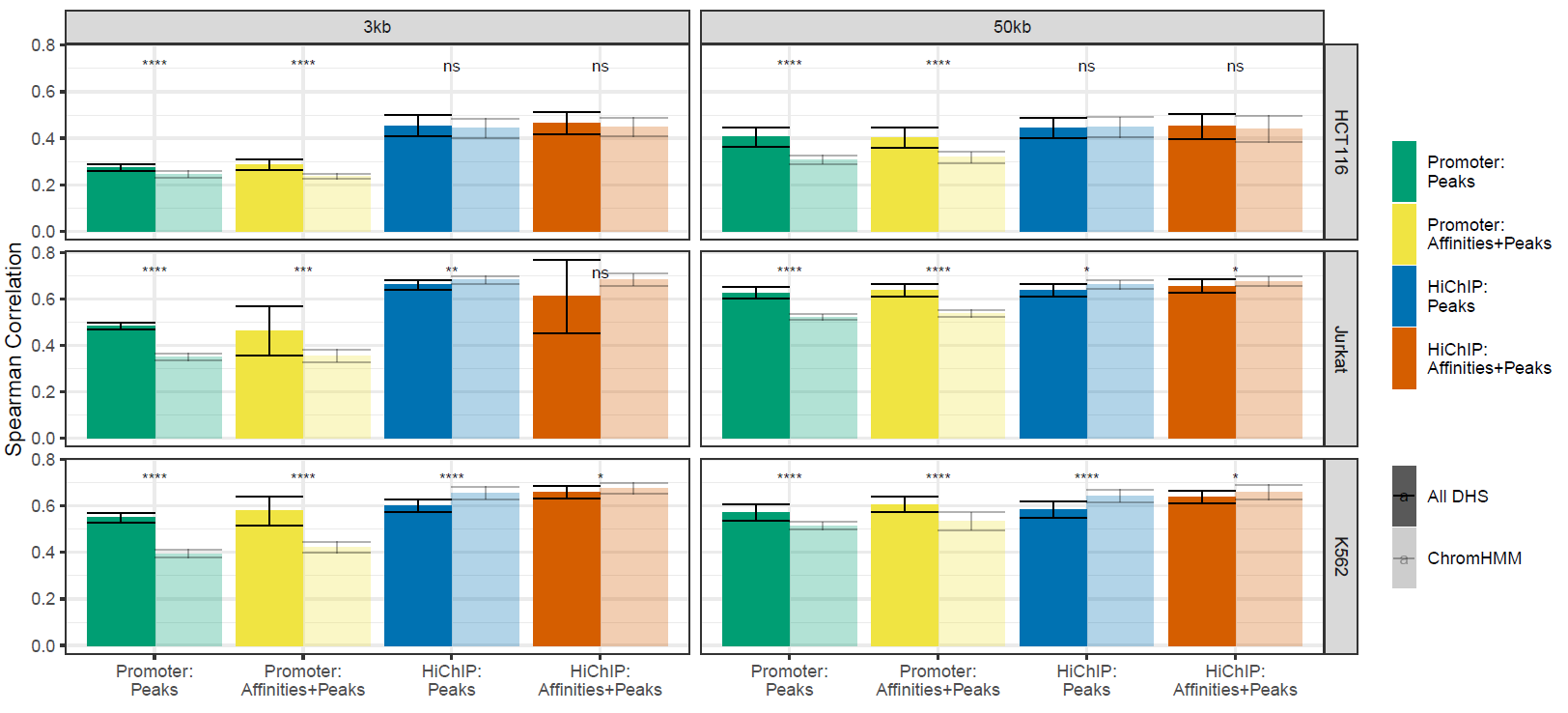


**Figure S18:** Performance of gene-expression models in terms of Spearman correlation, using peak and TF affinity features with and without ChromHMM filtering (ns: p ≥ 0.05, p < 0.05: *, p < 0.01: **, p < 0.001: ***, p < 0.0001: ****).


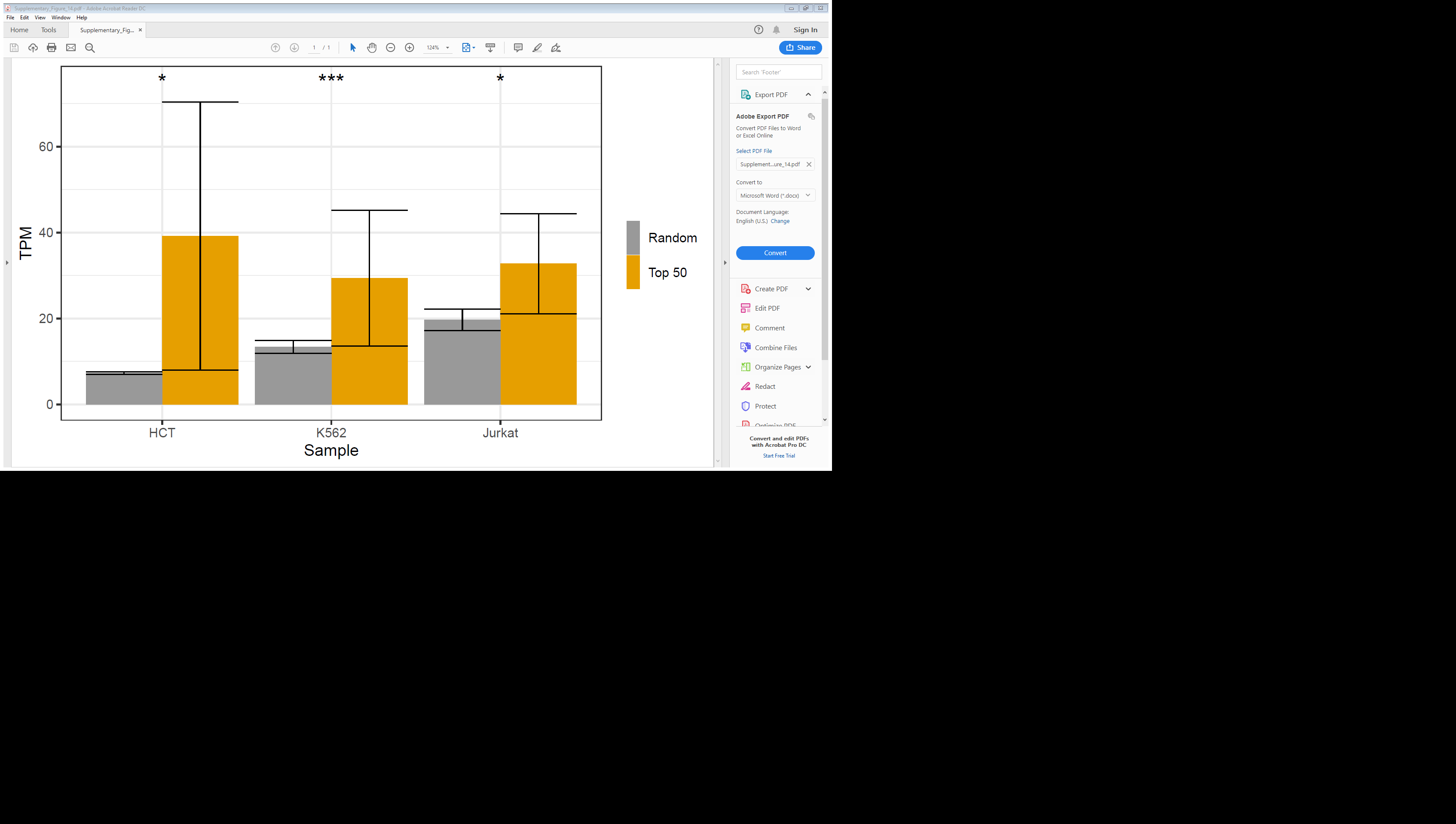


**Figure S19:** Expression of TFs measured in TPM for the top 50 TFs and 1000 randomly sampled sets of size 50 (ns: p ≥ 0.05, p < 0.05: *, p < 0.01: **, p < 0.001: ***).

# TEPIC HiC extension

We have extended the original TEPIC pipeline with a separate module, to incorporate chromatin conformation capture data, such as HiC and HiChIP data.The new modules incorporates so-called loop list files, which are produced for example by the *HiCCUPS* peak-calling algorithm.

However, we assured that the used format is very simplistic, such that any custom genomic contact information could be used as well. The files are tab separated stating the genomic position of the two loop sites in the following way:

***chr <tab> pos1 <tab> pos2 <tab> chr <tab> pos1 <tab> pos2 <tab> <track color> <tab> <contact counts>***

Aside from the loop list file, the HiC module offers a parameter controlling the loop window. It is used to identify loops that interact with the 5’ TSS of the gene of interest. Centered at the TSS, the implementation searches for genomic loci marking loop-sites in the loop list.

• **-h** If the name of the HiC loop ﬁle is provided, all open chromatin regions will be intersected with loop regions around the TSS of each gene.

The optional parameter is:

• **-s** Deﬁnes the size of the loop window [bp]. Default is 25000**.**

The double feature space TF affinities are computed automatically if the **-h** parameter is used, unless the **-q** parameter is set as well, which produces peak features only.
